# Flagellin acting via TLR5 is the major activator of key signaling pathways leading to NF-κB and proinflammatory gene program activation in intestinal epithelial cells

**DOI:** 10.1186/1471-2180-4-33

**Published:** 2004-08-23

**Authors:** Thomas Tallant, Amitabha Deb, Niladri Kar, Joseph Lupica, Michael J de Veer, Joseph A DiDonato

**Affiliations:** 1 Deparment of Cancer Biology, The Lerner Research Institute at the Cleveland Clinic Foundation, 9500 Euclid Avenue, Cleveland, OH 44195, USA; 2 Amitabha Deb, Massachusetts Biologics Labs, University of Massachusetts Medical School, 305 South Street, Jamaica Plain, MA 02130-3597, USA; 3 Michael de Veer, Centre for Animal Biotech, Department of Veterinary Science, Melbourne University, Parkville, Victoria, 3010, Australia

## Abstract

**Background:**

Infection of intestinal epithelial cells by pathogenic *Salmonella *leads to activation of signaling cascades that ultimately initiate the proinflammatory gene program. The transcription factor NF-κB is a key regulator/activator of this gene program and is potently activated. We explored the mechanism by which *Salmonella *activates NF-κB during infection of cultured intestinal epithelial cells and found that flagellin produced by the bacteria and contained on them leads to NF-κB activation in all the cells; invasion of cells by the bacteria is not required to activate NF-κB.

**Results:**

Purified flagellin activated the mitogen activated protein kinase (MAPK), stress-activated protein kinase (SAPK) and Ikappa B kinase (IKK) signaling pathways that lead to expression of the proinflammatory gene program in a temporal fashion nearly identical to that of infection of intestinal epithelial cells by *Salmonella*. Flagellin expression was required for *Salmonella *invasion of host cells and it activated NF-κB via toll-like receptor 5 (TLR5). Surprisingly, a number of cell lines found to be unresponsive to flagellin express TLR5 and expression of exogenous TLR5 in these cells induces NF-κB activity in response to flagellin challenge although not robustly. Conversely, overexpression of dominant-negative TLR5 alleles only partially blocks NF-κB activation by flagellin. These observations are consistent with the possibility of either a very stable TLR5 signaling complex, the existence of a low abundance flagellin co-receptor or required adapter, or both.

**Conclusion:**

These collective results provide the evidence that flagellin acts as the main determinant of *Salmonella *mediated NF-κB and proinflammatory signaling and gene activation by this flagellated pathogen. In addition, expression of the *fli C *gene appears to play an important role in the proper functioning of the TTSS since mutants that fail to express *fli C *are defective in expressing a subset of Sip proteins and fail to invade host cells. Flagellin added in *trans *cannot restore the ability of the *fli C *mutant bacteria to invade intestinal epithelial cells. Lastly, TLR5 expression in weak and non-responding cells indicates that additional factors may be required for efficient signal propagation in response to flagellin recognition.

## Background

Intestinal epithelial cells serve as a barrier between the luminal microflora and the body and as such are perfectly positioned to monitor the approach/invasion of pathogens. These intestinal epithelial cells (IECs) serve as innate immune sentinels and monitor their environment and constantly give out innate host defense instruction to local immune effector cells [1,2]. Pathogens such as *Salmonella *and other enteroinvasive pathogenic bacteria such as enteroinvasive *E. Coli*, *Shigella *and *Yersinia *upon infection of IECs leads to the up-regulation of the expression of host genes, the products of which activate mucosal inflammatory and immune responses and alter epithelial cell functions [3-6]. Previously we and others demonstrated that IKK via NF-κB and the SAPK signaling pathways via Jun N-terminal kinase (JNK) and p38 kinase were key regulators of the up-regulation of the proinflammatory gene program [3,7-9], with NF-κB appearing to be the most critical [3]. Typically *Salmonella *infects thirty-forty percent of IECs in culture models of infection [10], however, we and others have found that *Salmonella *infection activates NF-κB DNA binding activity to levels equivalent to that of TNFα which activates NF-κB in all of the cells [3]. Previous studies examining NF-κB activation by *Salmonella *in HT29 colonic intestinal epithelial cells, which serve as model colonic epithelial cells in culture, indicated that delivery of *Salmonella *proteins into the host cell via its type III secretion system (TTSS), such as SopE and SopE2, the bacterially encoded exchange factors for the Rho-family members Rac1 and CdC42, result in exchange factor activation, cytoskeletal rearrangements and activation of the MAPK, SAPK and NF-κB signaling pathways [7,8,11-15]. Recent observations that utilized *Salmonella *strains that were defective in invasion and delivery of invasion proteins by the TTSS but not attachment indicated that additional factors other than those delivered by the TTSS could lead to NF-κB activation [16]. Presently it is not clear what protein(s) dictate the activation of key signaling pathways that lead to the temporal expression of the proinflammatory gene program, although the SopE proteins have been given extreme attention recently [7,8,15].

In searching for additional *Salmonella *proteins that could activate the proinflammatory gene expression program, bacterial flagellin was recently found to be such a protein [16-19] and had been shown previously to activate IL-8 expression in monocytes [19-21]. Flagellin was found to activate NF-κB in polarized epithelial cells only when flagellin was present on their basolateral surface [22] consistent with the idea that a cell surface receptor was present there and could recognize it. The toll-like receptors (TLRs) have been found to recognize pathogen associated molecular patterns (PAMPs) reviewed in [23-26]. TLR2 interacts with TLR1 and TLR6 to recognize bacterial lipopeptides and zymosan respectively [27,28]. TLR4 recognizes LPS only when associated with its co-receptor MD2 and CD14 [29-32]. Recently, flagellin was demonstrated to be recognized by TLR5 and activate an innate host response [22,33]. However, little was known or demonstrated about the endogenous levels of TLR5 in cells used in those studies and why those cells failed to respond to flagellin. Here we have identified flagellin as the primary initiator and temporal regulator of not only the major signaling pathways activated during *Salmonella *infection but also of key target genes of the proinflammatory gene program too. We have also found flagellin expression to be required for *Salmonella *bacterial invasion. Independently we found TLR5 recognizes flagellin but its signaling activity toward this PAMP is consistent with either the aid of another flagellin-recognizing co-receptor (as TLR4 utilizes for LPS) or the use of another adapter protein, perhaps similar to MyD88, that is absent or present at low levels in flagellin non-or low-responding cells.

## Results

### Salmonella infection leads to a minority of cells invaded but activates NF-κB in nearly all cells

**Figure 1:**
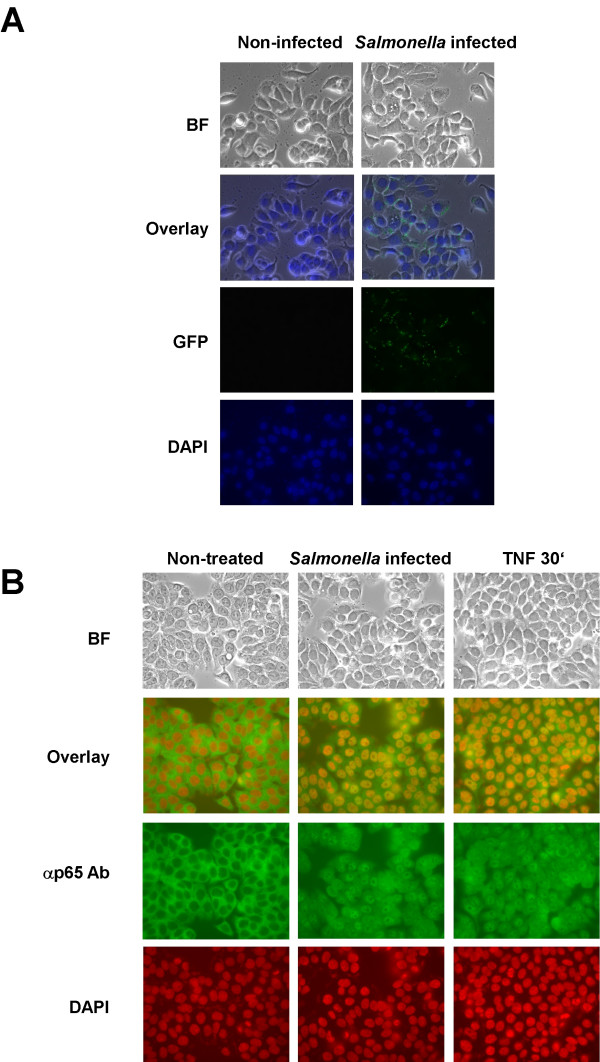
*Salmonella *infection leads to NF-κB nuclear localization even in non-infected cells. HT29 cells were grown on glass coverslips and either mock-infected, left untreated, infected with *Salmonella typhimurium*, or treated with TNFα (10 ng/ml). Cells fixed after 30 min (TNF) and 1 h (*Salmonella*) as described in Experimental Procedures and *Salmonella *that had invaded HT29 cells were detected by direct fluorescence microscopy of GFP expression, p65(RelA) localization was monitored by indirect immunoflourescence of rabbit anti-p65 antibody detected with FITC-conjugated donkey anti-rabbit antibody. DAPI was used to stain nuclei. A, HT29 cells were mock-infected or infected at an MOI of 50 with *Salmonella typhimurium *strain SJW1103G which expresses GFP from the ssaH promoter that is only active inside infected host cells [10,34]. Cells were photographed using bright field microscopy (BF), and immunoflourescence to detect GFP or DAPI staining as indicated. Images were merged (overlay) to reveal cells that were infected. B, HT29 cells were left untreated, infected with *Salmonella typhimurium *strain 1103 or treated with TNFα. NF-κB p65(RelA) localization under various conditions as indicated was monitored by indirect immunofluorescence. Cells were visualized by bright field microscopy (BF), cell nuclei were stained with DAPI and p65(RelA) was visualized with FITC. DAPI staining was falsely colored red to make visualization of the merge (overlay) easier to distinguish.

Previously, we have noted that pathogenic *Salmonella sp*. infection leads to potent IKK and NF-κB activation and activation of the proinflammatory gene program [3]. Previous studies suggest that about thirty-forty percent of the intestinal epithelial cells are infected during a typical *Salmonella *infection in cultured intestinal epithelial cells [10]. We wished to address the question of how bacterial infection of about thirty percent of the host cells could give rise to NF-κB DNA binding activity equivalent to activation of NF-κB in nearly all of the host cells as TNFα treatment of the cells does. To examine this phenomenon in detail HT29 cells either mock-infected or infected at a MOI of fifty for one-hour with wild-type *S. typhimurium *that had been transformed with the plasmid pFM10.1, that encodes green fluorescent protein (GFP) under the control of the *Salmonella *ssaH promoter and only functions once the bacteria has invaded the host cell [34]. As can be seen in Fig. 1A, GFP expression occurs in about thirty to forty percent of the cells. We next examined the localization of the NF-κB subunit p65 (RelA) in non-treated (mock-infected), *Salmonella *infected or TNFα (10 ng/ml) stimulated cells and found that p65 (RelA) was localized to the cytoplasm in non-treated cells whereas, in *Salmonella *infected cells or in TNFα treated cells p65 (RelA) had localized to the nucleus (Fig. 1B). These results demonstrate that *Salmonella *infection activates NF-κB in virtually all of the cells even though only a minority of them become infected and is consistent with and aids in explanation of our previous results examining *Salmonella *infection and NF-κB activation [3].

### Soluble bacterial product identified as flagellin can activate NF-κB in intestinal epithelial cells

Since *Salmonella sp*. infection of intestinal epithelial cells in culture led to only roughly thirty percent infection but activation of NF-κB in nearly all of the cells, we anticipated that NF-κB activation was in response to host cell recognition of bacteria structural components or products produced by the bacteria and not by the invasion process. Invasion itself has been demonstrated not to be required for activation of the proinflamatory gene program as had previously been thought [16]. To investigate this possibility sterile-filtered *S. dublin *culture broth left either untreated or boiled for twenty minutes was used to challenge HT29 intestinal epithelial cells and NF-κB DNA binding activity was monitored by electromobility shift assays (EMSAs) of whole cell extracts (WCE) prepared forty-five minutes after exposure [3,35]. Potent activation of NF-κB in response to the broth under both conditions was observed indicating the activating factor was heat-stable (AD, TT and JD, personal observations) and is not LPS since HT29 cells are not responsive to LPS [3,35].

**Figure 2:**
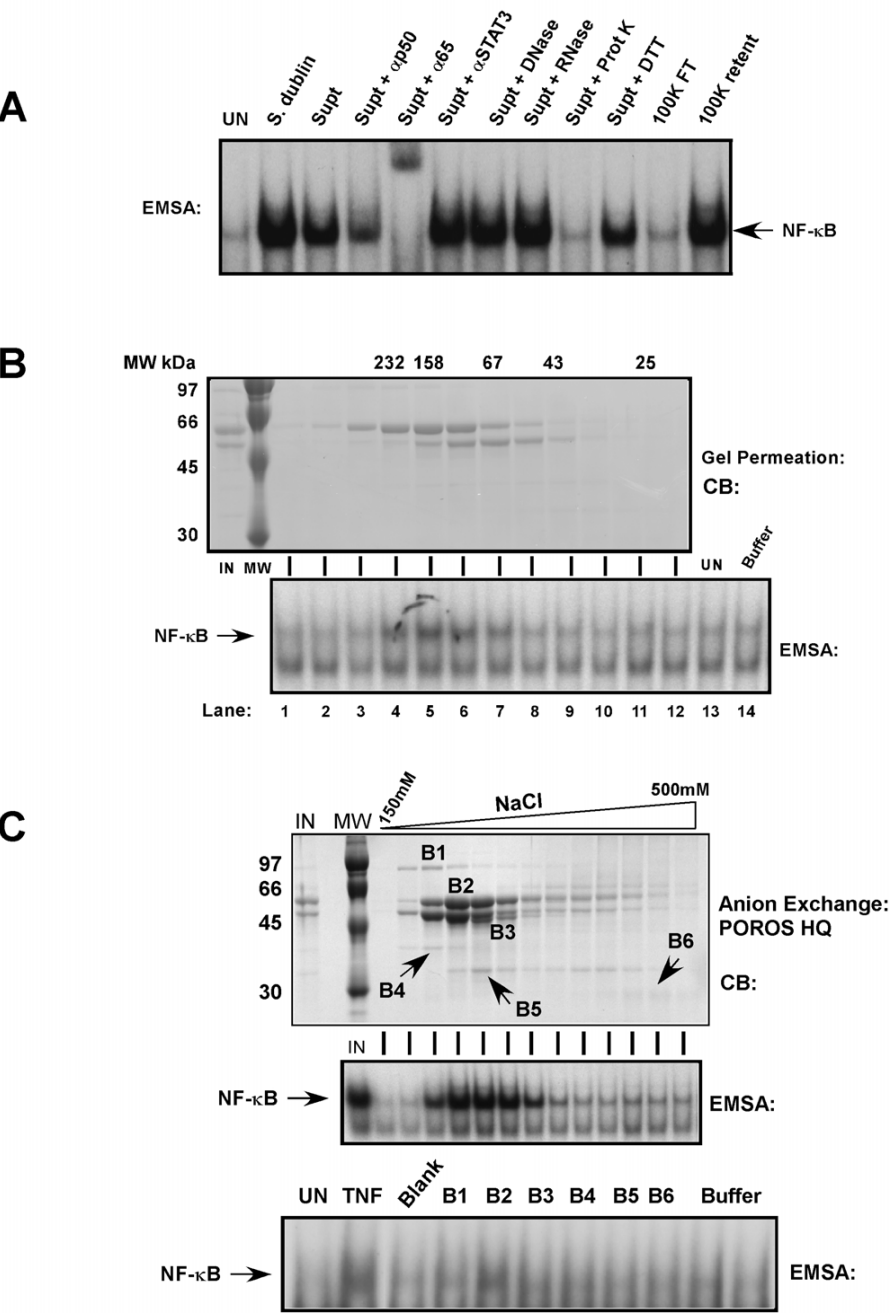
Protein factor in *Salmonella *culture broth leads to NF-κB activation. A, *Salmonella dublin *culture broth concentrated 100-fold was treated as indicated or infectious bacteria, as indicated was used to challenge HT29 cells. NF-κB DNA binding activity was assayed by EMSA from whole cell extracts prepared 45 min after treatment. Authenticity of the NF-κB DNA:protein complex was determined using p65(RelA)-specific and p50-specific antibody supershifts. B, Concentrated *Salmonella dublin *culture broth (IN) was chromatographed by gel permeation on a Superose 12 column. Eluted protein fractions were analyzed by fractionation on 10% SDS-PAGE and visualized by Coomassie blue (CB) staining. Molecular weight markers for chromatography and on the gels are indicated. Aliquots of each fraction as indicated was used to stimulate HT29 cells and resultant WCEs were analyzed by EMSA for NF-κB DNA binding activity. C, Concentrated *Salmonella dublin *culture broth (IN) was chromatographed by anion exchange chromatography on POROS HQ matrix. Proteins were eluted with an increasing NaCl gradient as indicated and analyzed on 10% SDS-PAGE and visualized by Coomassie blue (CB) staining. Input and aliquots of each fraction as indicated was used to stimulate HT29 cells and resultant WCEs were analyzed by EMSA for NF-κB DNA binding activity. Eluted material corresponding to protein bands B1-B6, a blank portion of the gel was isolated from a duplicate 10% SDS-PAGE gel as described in Experimental Procedures along with buffer samples from the beginning and end NaCl buffer gradient and used to stimulate HT29 cells and resultant WCEs were analyzed by EMSA for NF-κB DNA binding activity.

The native sterile-filtered concentrated broth was subsequently treated with DNase, RNase, proteinase K or crudely size fractionated on 100 kDa centricon filters. The variously treated broths were then used to challenge HT29 intestinal epithelial cells and WCEs were prepared after forty-five minutes and NF-κB DNA binding activity was analyzed by EMSA (Fig 2A). Direct infection of HT29 cells by *S. typhimurium *1103 or exposure to the culture broths (supt), as indicated, induced NF-κB DNA binding activity, while the activity-inducing factor was found to be sensitive to protease digestion and was retained by a 100 kDa filter (Fig. 2A). To further determine the identity of the NF-κB inducing activity, sterile-filtered concentrated broth culture was fractionated by Superose 12 gel permeation chromatography (Fig. 2B) and by anion exchange chromatography (Fig. 2C). Aliquots of chromatography fractions were assayed for their ability to activate NF-κB in HT29 cells and analyzed by EMSA. As can be seen from the Coomassie blue stained gel (Fig. 2B, top panel) increased NF-κB DNA binding activity (Fig. 2B, lower panel lanes 4–6) corresponded to the increased abundance of an approximately 55 kDa protein. Anion exchange chromatography on POROS HQ matrix and elution of bound proteins with an increasing salt gradient as indicated (Fig. 2C) demonstrated that NF-κB DNA binding-inducing activity corresponded to chromatographic fractions containing an increased abundance of the 55 kDa protein (Fig. 2C top panel, and data not shown). Eluted fractions observed in Fig. 2C were concentrated and fractionated on preparative 12% SDS-PAGE gels and bands corresponding to B1-B6 were cut from the gels and the proteins eluted, precipitated and renatured as described in Experimental Procedures and used to stimulate HT29 cells. Whole cell extracts from these cells were assayed for NF-κB DNA binding-inducing activity by EMSA and only band 2 (B2) corresponding to the 55 kDa protein (Fig. 2C lower panel) was able to elicit NF-κB DNA binding activity while buffer from the beginning or end of the salt gradient failed to activate NF-κB DNA binding activity.

**Figure 3:**
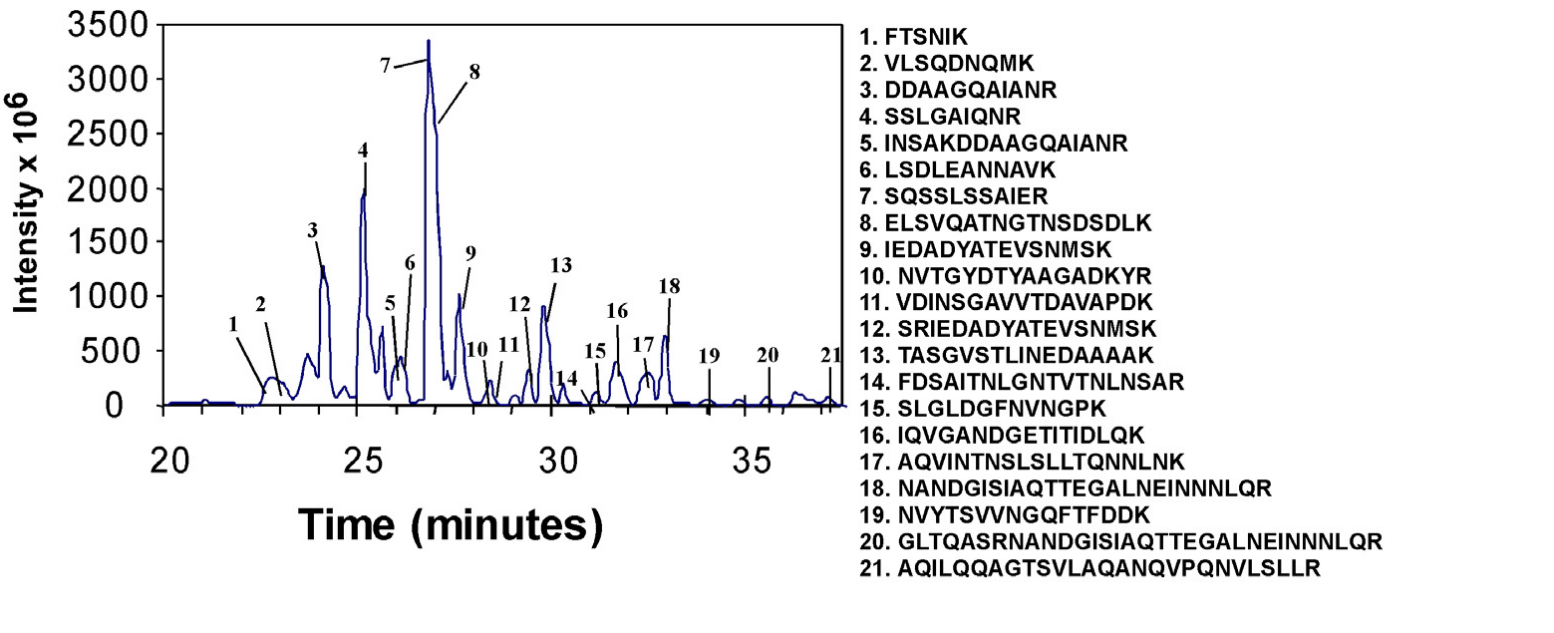
Identifcation by mass spectrometry of flagellin as the NF-κB activating factor in *Salmonella *culture broth. Microcapillary HPLC tandem mass spectrometry of Band 2 digested by trypsin. Peaks corresponding to *Salmonella *peptides are numbered and identified with the corresponding numbered peptide sequence to the right.

Proteins corresponding to protein bands B1-B6 and blank areas of the gel were further processed for peptide sequencing as described in Experimental Procedures. Trypsin digestion of the protein corresponding to B2 and analysis by electrospray ion trap LC/MS identified the amino acid sequence of twenty-one peptides. Flagellin (seventy-five percent coverage by the twenty-one peptides) was unambiguously identified as the protein consistent with inducing NF-κB DNA binding activity (Fig 3).

### Flagellin is required to activate NF-κB in intestinal epithelial cells

**Figure 4:**
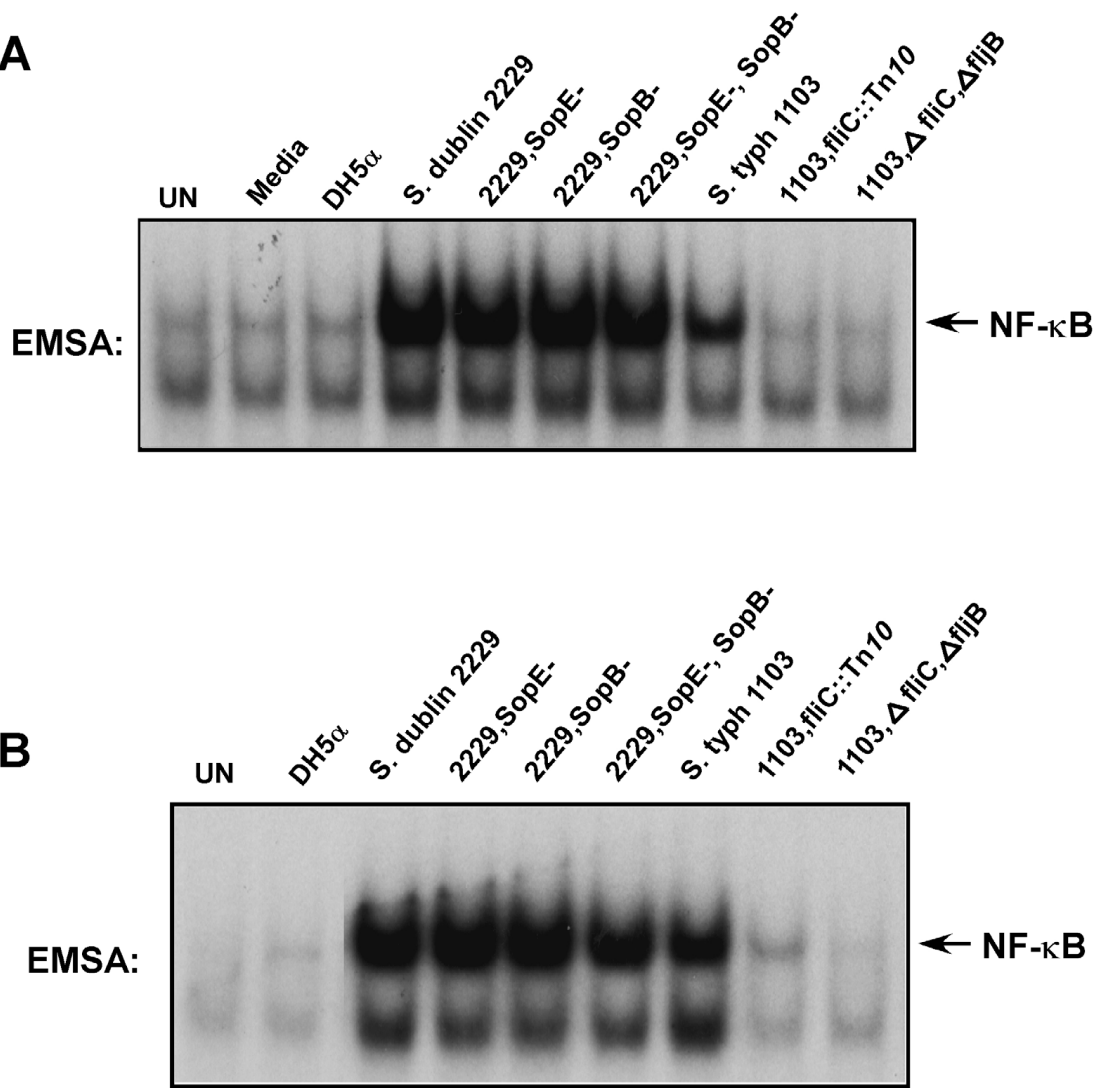
Flagellin mutants fail to activate NF-κB. EMSAs assaying for NF-κB DNA binding activity in WCEs prepared 45 min from non-infected cells (UN) and after direct infection of HT29 cells with wild-type *E. coli *DH5α, wild-type *Salmonella dublin *or SopE^- ^mutant, SopB^- ^mutant, the SopE^-^/SopB^- ^double mutant, wild-type *Salmonella typhimurium *strain 1103, the fliC^- ^mutant (fliC::Tn*10*), the fliC^-^/fljB^- ^double mutant as indicated at an MOI of 50. B, EMSAs assaying for NF-κB DNA binding activity in WCEs prepared 45 min after challenge of HT29 cells from non-infected cells (UN) or with sterile-filtered concentrated culture broths from wild-type and mutant bacteria as indicated.

To determine if flagellin was indeed the factor that was responsible for triggering activation of NF-κB after exposure of intestinal epithelial cells to direct bacterial infection or to filtered culture broths of pathogenic *Salmonella sp*. we prepared infectious bacteria and boiled and filtered culture broths from the non-flagellated E. Coli DH5α, pathogenic *S. dublin *strain 2229, an isogenic *S. dublin *2229 SopE^- ^mutant, isogenic *S. dublin *2229 SopB^- ^mutant, isogenic *S. dublin *2229 double SopE^-^/SopB^- ^mutant (strain SE1SB2), *S. typhimurium *strain 1103, and isogenic *S. typhimurium *fliC^::^Tn*10 *insertion mutant (strain 86) and a *S. typhimurium *1103 isogenic double mutant fliC^-^/fljB^- ^and were used to challenge HT29 cells. Bacteria and culture broths were used to challenge HT29 intestinal epithelial cells and WCE extracts were prepared after forty-five minutes and analyzed for NF-κB DNA binding activity by EMSA. *Salmonella *strains could activate NF-κB, while *Salmonella *strains failing to produce flagellin (fliC and fliC^-^/fljB^- ^mutants as indicated) also failed to activate NF-κB (Fig. 4A &4B). *E. Coli *DH5α is non-flagellated and does not produce flagellin failed to activate NF-κB. We also noticed through numerous experiments that *S. dublin *direct infections always activated NF-κB to a greater extent than *S. typhimurium *as observed in Fig. 4A while culture broths from both species activated NF-κB almost equally well (Fig. 4B). We believe this difference is due perhaps to *S. dublin *releasing more flagellin into the cell culture media than *S. typhimurium *during infection since purification of flagellin from both *S. dublin *and *S. typhimurium *and addition of equivalent amounts of chromatographically purified flagellin gave similar NF-κB activation profiles (TT & JD, unpublished observations).

Of note is the total failure of the double flagellin gene mutants to activate NF-κB as compared to the very minor activation observed in the single Phase I flagellin fliC::Tn*10 *insertion mutant (next to last lanes in Fig. 4A &4B) which likely is due to the extremely limited expression of the phase II flagellin (from fljB), although the strains of *Salmonella *used here genetically are unable or rarely shift phases of flagellin production. These results are consistent with previous reports identifying flagellin as a potent inducer of the proinflammatory response and IL-8 production [16-19]. Since flagellin appears required for activation of the NF-κB pathway upon direct infection of intestinal epithelial cells it appeared possible that flagellin may also be the major determinant of other major mitogenic and stress activated signaling pathways activated upon pathogenic *Salmonella *infection of intestinal epithelial cells. Previously others and we have demonstrated that direct *Salmonella *infection of intestinal epithelial cells results in JNK activation [8] and also the activation of NF-κB via IKK [3]. The identification of flagellin as a potent NF-κB activator is significant since SopE had previously been shown to be a pathogenic *Salmonella *bacteriophage encoded protein that is injected into the host cell and acts as an exchange factor for the small Rho GTPases Rac1 and CdC42 initiating cytoskeleton rearrangements and eventual activation of the MAPK, SAPK and NF-κB pathways [7,15], while SopB is a *Salmonella *protein that functions as an inositol phosphate phosphatase and participates in cytoskeletal rearrangements and stimulates host cell chloride secretion [36].

### Flagellin triggers activation of the mitogen activated protein kinase, stress activated protein kinase and IKK signaling pathways

**Figure 5:**
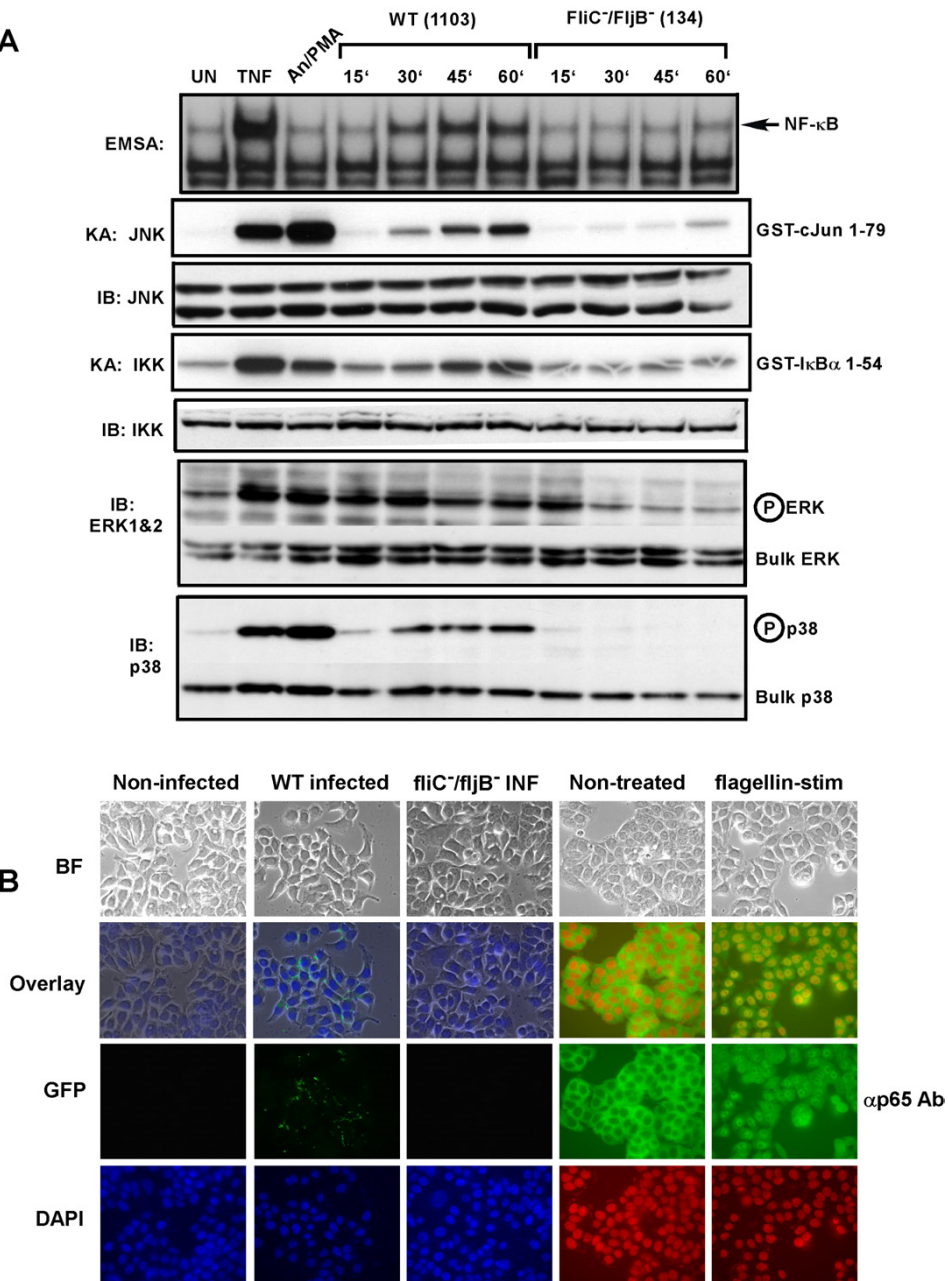
Flagellin is required for activating multiple signaling pathways during *Salmonella *infection and leads to nuclear localization of NF-κB. HT29 cells were left untreated, stimulated with TNFα (10 ng/ml) or a cocktail of anisomycin [An] (20 μg/ml)/PMA (12.5 ng/ml) for 15 min, or infected with either wild-type (WT) *Salmonella typhimurium *strain 1103 or the *Salmonella typhimurium *double fliC^-^/fljB^- ^mutant strain 134 as indicated. WCE were prepared at the indicated times or at 10 min for TNF-treated cells or 15 min for anisomycin/PMA treated cells and used in EMSAs to analyze NF-κB DNA binding activity, or in immuno-kinase assays (KA) using anti-IKK or anti-JNK antibodies to measure IKK and JNK kinase activity on their respective substrates GST-IκBα 1–54 and GST-cJun 1–79 (as indicated). Immunoblot (IB) analysis of equivalent amounts (40 μg) of protein from each extract was fractionated on SDS-PAGE gels and transferred to PVDF membranes and probed with the indicated antibodies to detect bulk IKK, JNK, ERK and p38 as indicated. Immunoblot analysis using phospho-specific antibodies for ERK and p38 to detect activated ERK and p38 are indicated. B, Immunofluorescence demonstrating that flagellin mutant Salmonella fail to infect HT29 cells and that purified flagellin stimulation of HT29 cells leads to NF-κB nuclear p65 (RelA) localization as determined by indirect immunofluorescence. Imaging of the treatment indicated HT29 cells grown on coverslips was essentially the same as in Fig. 1A & 1B. False coloring of the DAPI stain was used to enhance the visualization of both DAPI stained nuclei and p65 nuclear localization.

Intestinal epithelial cells act as sentinels for invasion of luminal surfaces and orchestrate the attraction of effector immune cells to the area by production of chemokine genes like IL-8 and macrophage chemoattractant protein 1 (MCP1) proinflammatory cytokine genes such as TNFα, IL-1 and IL-6 [1,4-6]. Expression of these genes primarily depends upon the action of transcription factors that are activated in response to the transmission of signals via the MAPK, SAPK and IKK signaling pathways. Since NF-κB is considered a central regulator/activator of the proinflammatory gene program we decided to examine the effect that non-flagellin producing mutant strains of *Salmonella *had on activation of the MAPK, SAPK and IKK signaling pathways compared to infection of intestinal epithelial cells with wild-type *Salmonella *or by exposure of the intestinal epithelial cells to purified flagellin. Infection of HT29 cells with wild-type *S. typhimurium *resulted in activation of MAPKs ERK1&2, the SAPKs p38, JNK and IKK (Fig. 5) as determined by use of activation-indicating phospho-specific antibodies in immunoblot (IB) analysis or antibody-specific immuno-kinase assays (KA) for JNK and IKK using their respective substrates GST-cJun 1–79 and GST-IκBα1–54 [37-39]. Interestingly, MAPK stimulation is transient in nature as activation declines beginning at forty-five minutes while p38, JNK and IKK activity increases with time through one hour. As seen in Fig. 4, the fliC^-^/fljB^- ^double mutant *Salmonella *also failed to induce IKK and NF-κB activity (Fig. 5 as indicated). Surprisingly, the fliC^-^/fljB^- ^double mutant *Salmonella *failed to induce the SAPKs p38 and JNK and only briefly (fifteen minutes) activated MAPK. This result is puzzling since other *Salmonella *proteins such as SopE and SopE2 can activate the small GTPases Rac and CdC42, and these Rho family GTPases have been linked to JNK and p38 activation [7,8,14,15] yet appear not to function in the flagellin minus strain.

The fliC^-^/fljB^- ^double mutant *Salmonella *failed to invade HT29 cells compared to the wild-type *Salmonella *strain as determined by gentamycin protection/invasion assay (see Experimental Procedures). The flagellin fliC^-^/fljB^- ^double mutant displayed a four orders of magnitude difference in its ability to invade HT29 cells (TT & JD, unpublished observations). To demonstrate this point further, we infected HT29 cells with either wild-type *Salmonella *or the fliC^-^/fljB^- ^double mutant *Salmonella *(strain 134), both strains were transformed with the plasmid pFM10.1 that encodes GFP under the control of the Salmonella ssaH promoter and only functions once the bacteria has invaded the host cell [10,34]. The wild-type *Salmonella *clearly was able to infect HT29 cells (GFP, Fig. 5B) while the flagellin mutant bacteria failed to invade HT29 cells as evidenced by the lack of GFP expression (Fig. 5B). To determine if flagellin is sufficient or that other bacterially produced proteins are required for invasion, we added either purified flagellin or sterile-filtered culture broths or a combination of both to HT29 cells that were challenged with the *Salmonella *fliC^-^/fljB^- ^double mutant and assayed for invasion. Intestinal epithelial cells failed to be invaded using all tested combinations of purified flagellin and/or culture broths with the fliC^-^/fljB^- ^double mutant strain (TT & JD, unpublished observations). To our knowledge there is no known direct connection between expression of flagellin genes and the effectiveness of the type III secretion system to deliver bacterially produced proteins such as SopE, SopE2 and SipA or other Sip or Sop proteins [7,14,15,40,41] that play important roles in initiating bacterial internalization. Furthermore, to evaluate the effectiveness of flagellin to stimulate p65 (RelA) nuclear localization in intestinal epithelial cells we challenged HT29 cells with purified flagellin and examined p65 (RelA) localization using indirect immunofluorescence and found p65 (RelA) nuclear localization in nearly every cell (Fig. 5B as indicated).

**Figure 6:**
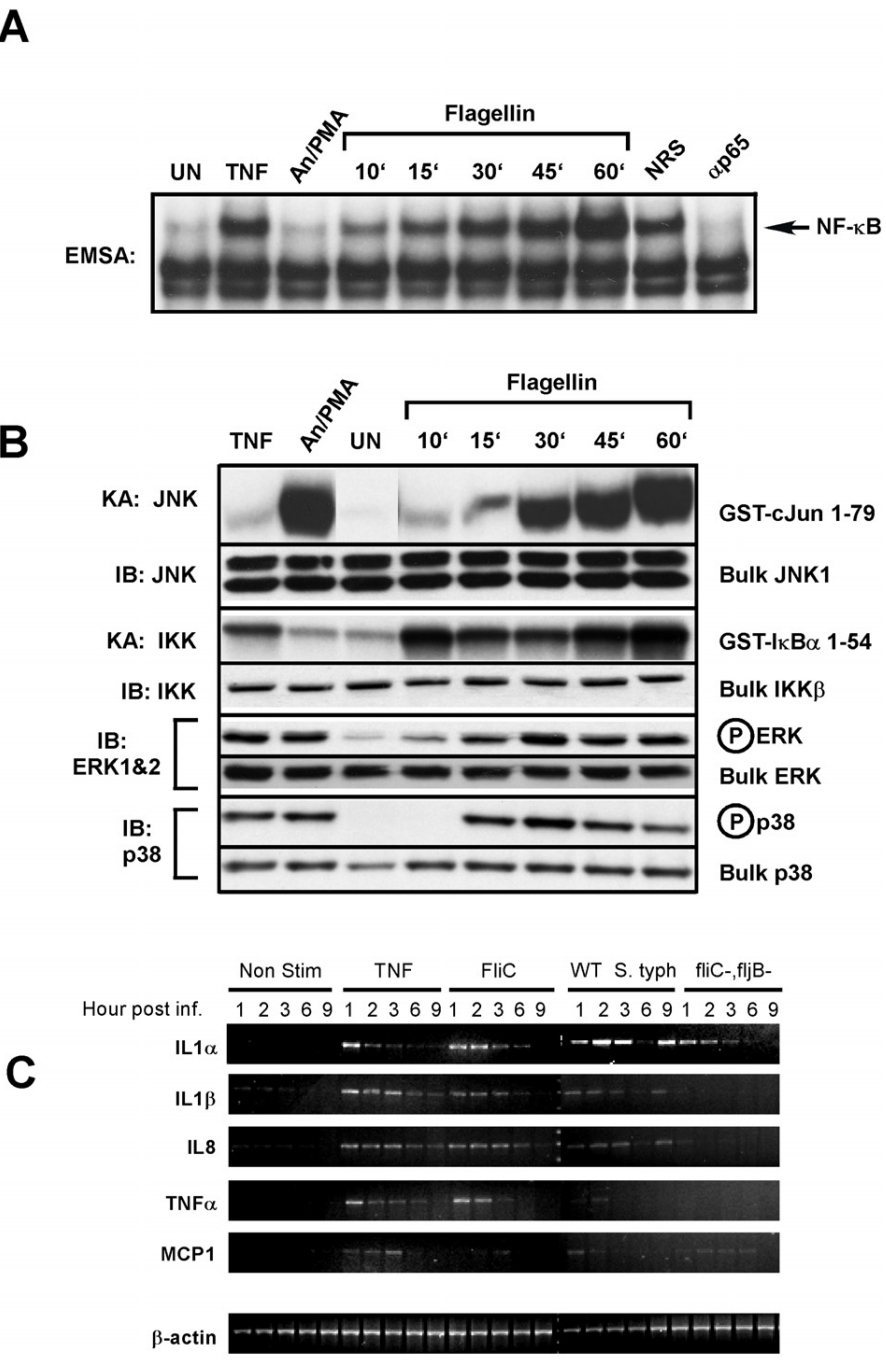
Purified flagellin activates signaling pathways and proinflammatory gene expression in intestinal epithelial cells mimicking that of wildtype a wild-type *Salmonella *infection. HT29 cells were left untreated or treated with TNFα (10 ng/ml) or a cocktail of anisomycin [An] (20 μg/ml)/PMA (12.5 ng/ml) for 10 min, or with flagellin (1 μg/ml) for the indicated times. WCE were prepared and analyzed by EMSA for NF-κB DNA binding activity, immuno-kinase assays (KA) or immunoblot analysis using phospho-specific antibodies for ERK or p38 to detect activation and with kinase-specific antibodies as described in Fig. 5A to detect bulk kinase abundance as indicated. A, EMSA to detect NF-κB DNA binding activity. Authenticity of the NF-κB bandshift was tested with supershift of the complex with p65(RelA)-specific antibody (α p65), normal rabbit serum (NRS) served as an irrelevant antibody control. B, immunoblot and kinase assays to detect IKK, JNK, ERK and p38 kinase activities and protein abundance as in Fig. 5A. C, semi-quantitative RT-PCR of proinflammatory gene expression of non-treated, wild-type and flagellin double mutant Salmonella typhimurium infected, TNFα (10 ng/ml) or flagellin (1 μg/ml) stimulated cells. HT29 cells were harvested at the indicated times after the indicated treatments and isolated RNA was used to make first strand cDNA that subsequently used in RT-PCR reactions (as described in Experimental Procedures) using gene-specific primers for IL1α, IL1β, IL-8, TNFα, MCP1 and β-actin. β-actin was used as a standard for normalizing expression patterns. Resulting PCR products were fractionated on 2% agarose gels and visualized by eithidium bromide staining.

Purified flagellin (0.5 μg/ml) was added to the culture media of HT29 cells and WCE were prepared at various times as indicated after exposure and assayed for NF-κB DNA binding activity in EMSAs (Fig. 6A). Flagellin potently activated NF-κB in a time dependent manner similar to that observed for TNF (10 ng/ml) treatment of HT29 cells (Fig. 6A). Analysis of the MAPK, SAPK and IKK signaling pathways (Fig. 6B) at various times after flagellin treatment of HT29 cells using activation-specific phospho-antibodies to monitor MAPK and p38 kinase activation or antibody-specific immunoprecipitation kinase assays for JNK and IKK activities demonstrated that JNK and IKK activity increased through time to one-hour while p38 and MAPK (ERK1&2) activity peaked at thirty minutes and began to decline to noticeably lower levels by one-hour (Fig 6B as indicated). The activation profile of the MAPK, SAPK and IKK signaling molecules ERK1&2, p38, JNK and IKK in intestinal epithelial cells in response to purified flagellin exposure remarkably resembled that of intestinal epithelial cells infected with wild-type *Salmonella *(Fig. 5A). From these observations we conclude that the temporal activation of the signaling pathways examined here (MAPK, SAPK and IKK), which reflect early events in *Salmonella *infection, are determined almost exclusively by recognition and response of intestinal epithelial cells to flagellin.

We wished to further examine the effect of purified flagellin and flagellin present on *Salmonella *on the temporal pattern of proinflammatory cytokine gene expression in intestinal epithelial cells in order to differentiate the effects of flagellin alone vs. flagellated *Salmonella *or non-flagellated *Salmonella *infection. HT29 cells were left untreated, stimulated with TNFα (10 ng/ml), or stimulated with flagellin (0.5 ug/ml), or infected with wild-type *Salmonella *typhimurium or the *Salmonella *fliC/fljB double mutant (at MOI of 50). After the indicated times after treatment or infection, HT29 cells were harvested in ice-cold PBS and the cell pellets lysed in Trizol and RNA was purified and used to prepare first-strand cDNA (see Experimental Procedures). Aliquots of the cDNA were used in semi-quantitative RT-PCR reactions using IL1α, IL-1β, IL-8, TNFα, MCP1 and β-actin gene specific primers (sequences available upon request) and the products were fractionated on ethidium bromide containing 1.2% agarose gels. Expression of the known NF-κB target genes IL-1β, IL-8, TNFα and MCP1 was increased in response to TNFα or purified flagellin exposure (Fig. 6C). Wild-type *Salmonella *infection also led to activation of these same genes although the expression of TNFα and MCP1 was transient in comparison and occurred immediately after infection. The *Salmonella *fliC^-^/fljB^- ^double mutant failed to induce IL-1β, IL-8 and TNFα expression, however MCP1 expression was induced, although at lower levels than that induced by wild-type *Salmonella*, and also, the expression of MCP1 was not transient in nature and continued throughout the time course (9 h) (Fig. 6C). The expression level of β-actin served as an internal standard for comparison. Interestingly, IL-1α, which is not an NF-κB target gene was stimulated in response to HT29 cell challenge by all of the treatments. Obviously, the *Salmonella *fliC^-^/fljB^- ^double mutant can activate other signaling pathways leading to IL-1α expression. We presently do not know what these signaling pathways are.

### Flagellin activates NF-κB DNA binding in a MyD88-dependent manner

**Figure 7:**
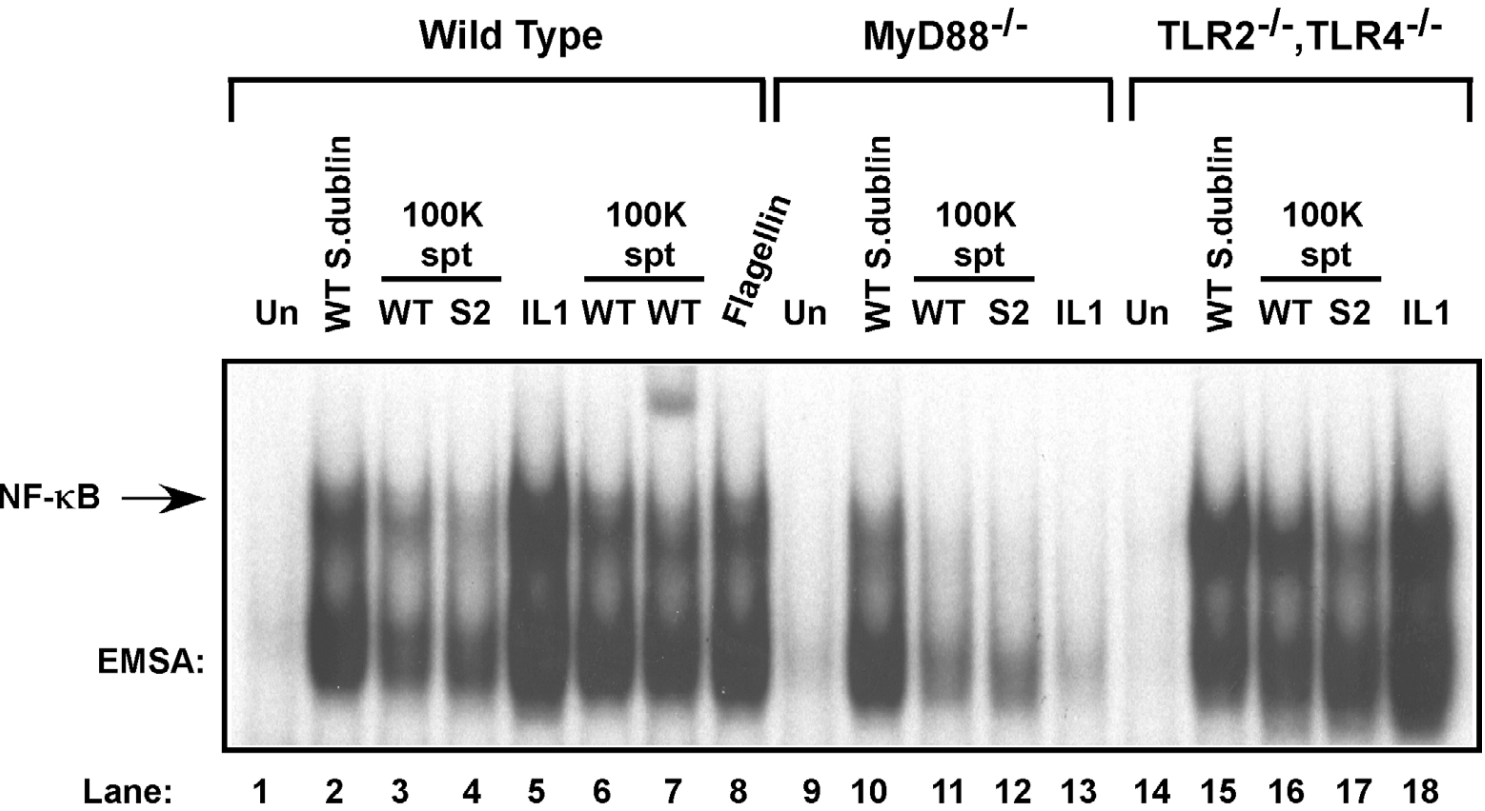
Flagellin-mediated activation of NF-κB is MyD88 dependent. Infectious wild-type *Salmonella Dublin *(MOI of 100), IL-1 (20 ng/ml), purified flagellin (1 μg/ml) (as indicated), sterile-filtered and concentrated 100 kDa filter retentate supernatant (spt) from wild-type *Salmonella dublin *and SopE^-^/SopB^- ^double mutant *Salmonella dublin *strain SE1SB2 (S2, as indicated) was used to challenge wild-type, MyD88^-/- ^knockout or TLR2^-/-^/TLR4^-/- ^double knockout MEFs as indicated. WCEs were prepared 45 min after treatments and examined by EMSA to analyze NF-κB DNA binding activity. IL-1 (20 ng/ml) was used as a positive control to monitor MyD88 function.

Flagellin was capable of activating the requisite signaling pathways consistent with proinflammatory gene activation similar to that of a cytokine like TNFα that activates all cells on which a functional cell surface receptor for it is present (see p65 [RelA] nuclear localization in Fig. 1 and Fig. 5C) we decided to examine the potential of the Toll-like receptors, to activate the NF-κB pathway in response to flagellin exposure. To quickly test this hypothesis we examined the effect that a dominant-negative MyD88 (aa 152–296) [42] expressing adenovirus had on flagellin-mediated NF-κB activation in HT29 cells. MyD88 is an adapter protein utilized by the IL-1 receptor and all of the known TLRs, which share homology to IL-1 through their cytoplasmic signaling domain and is required for immediate activation of the NF-κB pathway [43,44]. We found that expression of DN-MyD88 in HT29 cells blocked the activation of NF-κB DNA binding activity assayed by EMSA analysis in response to IL-1 or flagellin exposure, consistent with the action of a TLR-mediated activation of NF-κB (TT & JD, unpublished observations). To examine this possibility further we initially used wild-type, MyD88^-/- ^and TLR2^-/-^/TLR4^-/- ^MEFs (a gift of S. Akira, Univ. of Osaka, JA) to verify the role of MyD88 and to examine the potential role of two of the TLRs to respond to flagellin or to direct wild-type *Salmonella *infection and lead to NF-κB activation (Fig. 7). Wild-type *Salmonella *infection activates NF-κB potently in both the wild-type and TLR deficient MEFs (lanes 2 & 15) but this activation is somewhat defective in the MyD88 deficient MEFs (lane 10). Challenge of all three types of cells with concentrated sterile-filtered wild-type *S. dublin *or the double SopE^-^/SopB^- ^isogenic mutant *S. dublin *strain SE1SB2 culture broths activated NF-κB in wild-type MEFs and TLR2/4 double deficient cells but failed to activate NF-κB in MyD88 deficient cells (compare lanes 11 and 12 with lanes 3, 4, 6, 7, 16 and 17). NF-κB was potently activated in wild-type MEFs by exposure to purified flagellin (0.5 μg/ml) and therefore eliminated the possibility that LPS played a role in NF-κB activation in these experiments. The exclusion of LPS as a major contributor to NF-κB activation is also provided by the potent activation of the TLR2/4 double deficient MEFs (lanes 16 & 17). TLRs 2 and 4 respond to bacterial lipopeptides, peptidoglycans, certain LPSs and gram negative LPS respectively [45-47]. IL-1 stimulation verified the functional requirement of MyD88 in transmission of IL-1 and flagellin-mediated signals.

**Figure 9:**
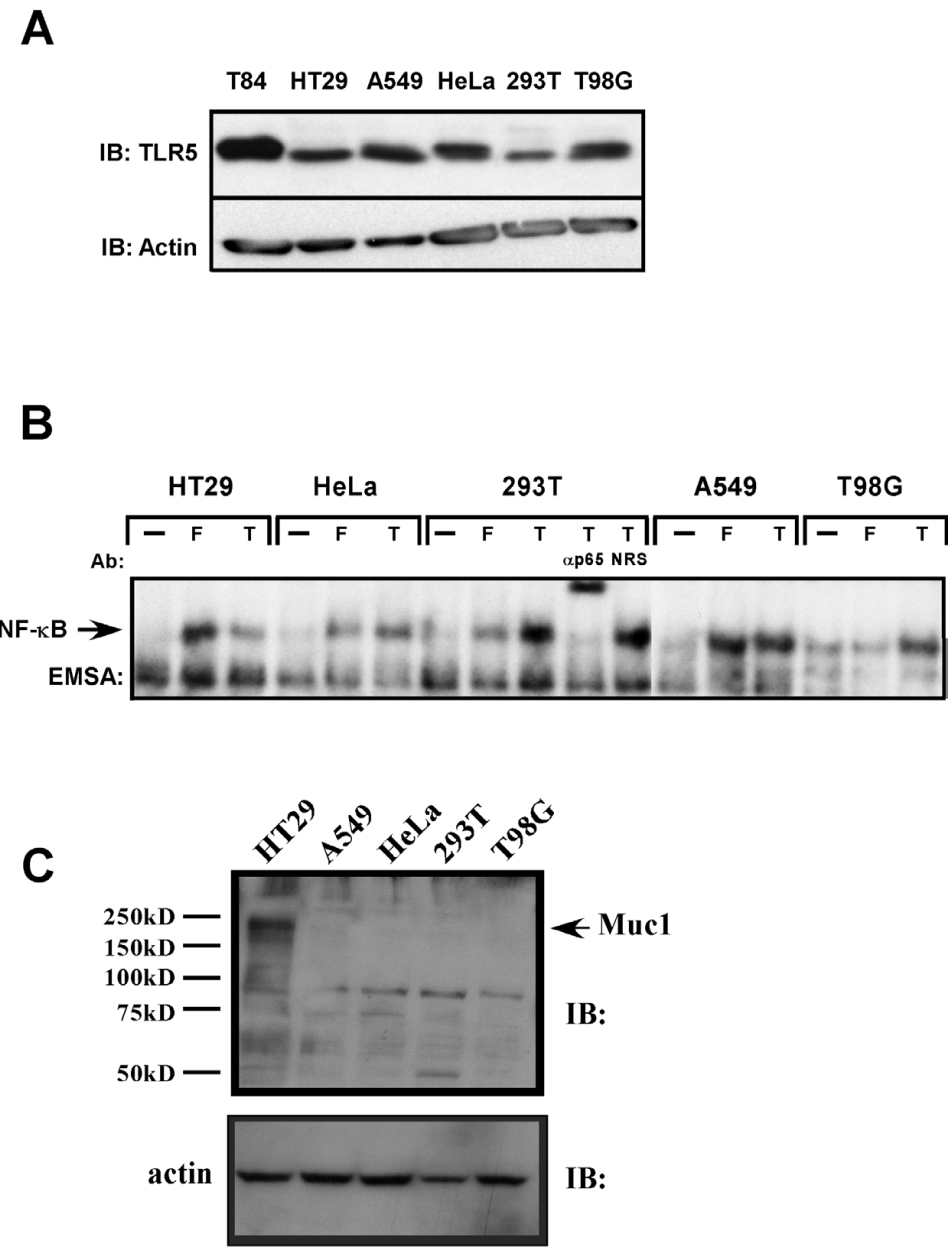
TLR5 is expressed in numerous cell types and has variable responses to flagellin. A, whole cell extracts were prepared from non-stimulated T84, HT29, A549, HeLa, 293T and T98G cells and fractionated on a 8% SDS-PAGE gel, proteins were transferred to PVDF membrane and probed with anti-TLR5 antibody for immunoblot analysis (IB). Protein loading was examined by probing with anti-actin antibody. B, HT29, A549, HeLa, 293T and T98G cells were left untreated (--), treated with flagellin (F) or TNFα (T) and WCEs were prepared after 45 min and used in EMSA to monitor NF-κB DNA binding activity. Authenticity of the NF-κB bandshift was tested with supershift of the complex with p65(RelA)-specific antibody (αp65), normal rabbit serum (NRS) served as an irrelevant antibody control. C, HT29, A549, HeLa, 293T and T98G cells WCEs (50 μg) were fractionated on a 8% SDS-PAGE gel, proteins transferred to Immobilon P and immunoprobed with anti-muc1 (1:450, Santa Cruz). Size markers are listed and muc1 position is indicated with an arrow.

To further define a possible role for the TLRs in flagellin recognition we assayed for the ability of overexpressed TLRs to activate NF-κB in cells that normally respond poorly to flagellin exposure. Choosing cells that responded slightly to purified flagellin ensured that the signaling components and adapters that flagellin uses were present and functional and that the limiting factor was likely only to be the receptor that responds to flagellin. We found that HeLa cells and HEK293T cells activated NF-κB DNA binding activity in response to IL-1 stimulation but poorly to flagellin exposure (TT & JD, unpublished observations) (but see Fig. 9B) and we chose HEK293T cells to use further because of their greater transfection efficiency. Amino-terminus FLAG epitope-tagged TLRs 1–9 (kind gifts of R. Medzhitov, Yale Univ. and R. Ulevitch, TSRI) [48,49] were overexpressed in HEK 293T cells in transient transfections along with the 2×-NF-κB-dependent promoter driven luciferase reporter gene [50] and the expression of luciferase in response to no treatment, flagellin (0.5 μg/ml) or TNFα (10 ng/ml) was determined. TLR5 was the only TLR whose expression resulted in a noticeable response to flagellin challenge of the cells (Table 1).

**Table 1:** *TLR*5 reponds to flagellin and activates NF-κB

	**No Stim**	**TNF**	**Flic**
Vector	1	13.5	4.9
TLR1	1.7	ND	5.1
TLR2	1.6	ND	5.3
TLR3	1.5	ND	5.0
TLR4	1.8	ND	5.4
TLR5	1.6	ND	9.2*
TLR7	1.5	ND	5.2
TLR8	1.4	ND	5.0
TLR9	1.5	ND	5.1

293T cells were transfected with empty vector (pCDNA3.1) or the individual listed wild-type TLR alleles in triplicate in 6-well dishes. Cells were left untreated (No Stim), TNFα (10 ng/ml) or flagellin (1 μg/ml). NF-κB reporter activity was adjusted by normalizing expression to control Renilla luciferase activity and fold induction was calculated as reporter gene activity in treated cells/reporter gene activity in non-stimulated cells. ND is not determined.

**Figure 8:**
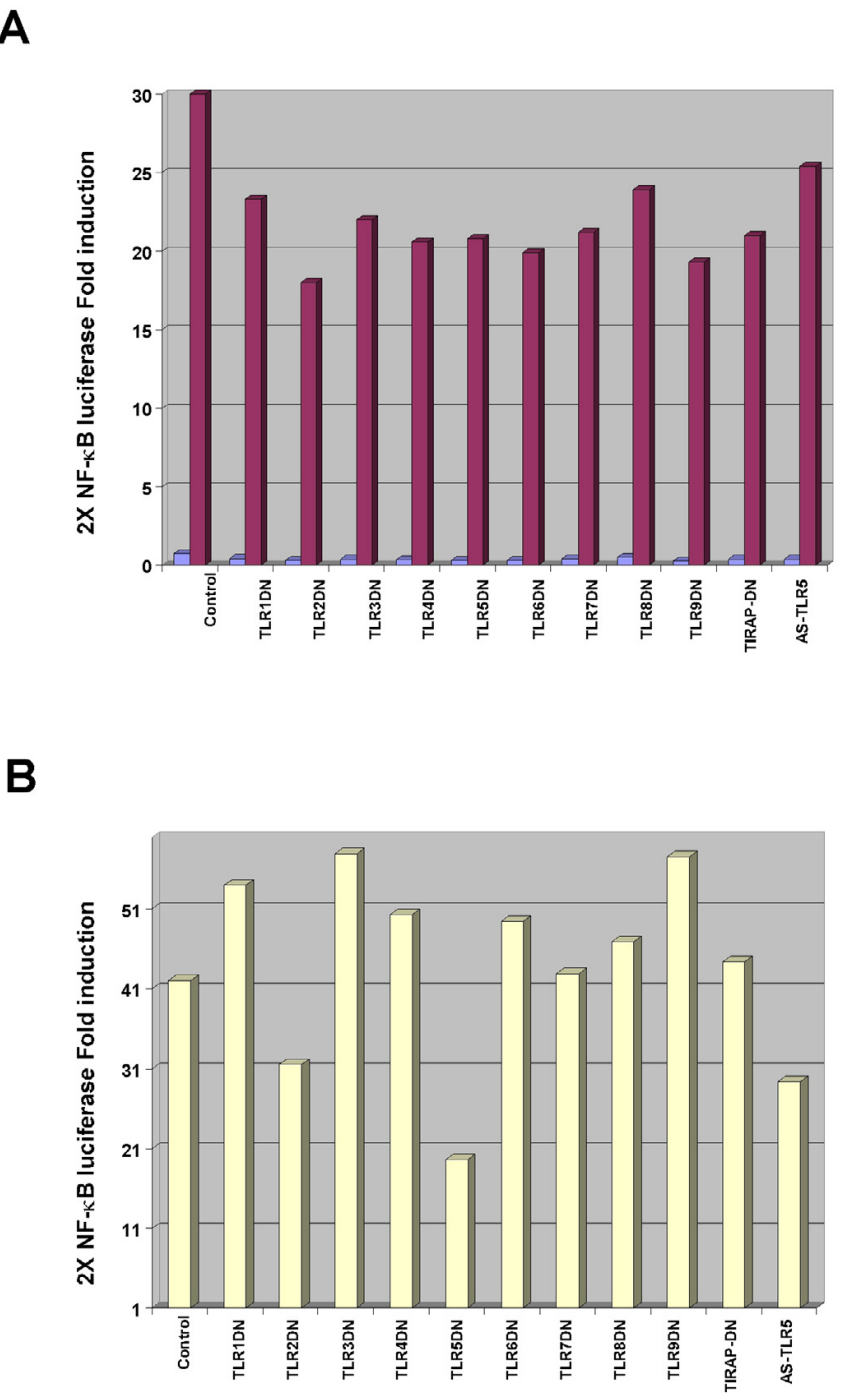
TLR5 inhibits flagellin-mediated NF-κB reporter gene activity. HT29 cells were transfected in triplicate in 6-well dishes using the indicated DN-TLR mammalian expression vectors or antisense TLR5 (AS TLR5) (2 μg/well), 2× NF-κB Luc reporter gene (100 ng/well), pRL-TK Renilla luciferase for normalization (50 ng/well) adjusted to 4 μg total DNA/well with empty vector pCDNA3.1 DNA. A, Fold-induction of 2× NF-κB Luc reporter gene in non-stimulated cells (light shading) and in TNFα (10 ng/ml) treated cells (dark shading). Lysates were prepared 12 h after stimulation. Results of a representative experiment are shown. B, HT29 cells transfected as in A were treated with flagellin (1 μg/ml) and cell lysates were prepared and analyzed as in Fig. 8A. Results of a representative experiment are shown.

To further determine the likelihood of TLR5 being the TLR through which flagellin activated NF-κB, we constructed dominant-negative signaling mutations by deletion of the carboxyl portion of each TLR to a conserved tryptophan in the TIR domain (see Materials and Methods). A similar mutation in the IL-1 receptor abrogates its ability to lead to NF-κB activation [51,52]. Each DN-TLR along with a reverse cloned TLR5 (AS-TLR5) were cloned into the mammalian expression vector pCDNA3.1 (Invitrogen, Carlsbad, CA). All mutant proteins were expressed well (TT & JD unpublished observations). Each DN-TLR mammalian expression vector and empty expression vector along with 2× NF-κB Luc was transfected as previously described [3] into HT29 cells which respond very well to flagellin. The transfected cells were left untreated, stimulated with TNFα (10 ng/ml) or with purified flagellin (0.5 μg/ml). Reporter gene expression was observed not to be affected by DN-TLR expression in response to TNFα stimulation of transfected cells (Fig. 8A) however, only expression of either the DN-TLR5 or an antisense TLR5 construct resulted in a nearly fifty percent and twenty-five percent inhibition of flagellin-mediated reporter gene activation respectively (Fig. 8B), while DN-TLR2 also was found to mildly inhibit flagellin-mediated reporter expression. These results imply that TLR5 takes part in cell surface recognition of flagellin and initiates the signaling pathway leading to NF-κB activation. The effect of DN-TLR2 on NF-κB-dependent reporter gene activation may be non-specific since its expression also inhibited TNFα-mediated reporter activation as compared to the other DN-TLRs. DN-TLR2 may also compete for an unknown adapter protein that both TLR2 and TLR5 might share. In any event, TLR2 and TLR4 were shown by the results presented in Fig. 7 not to be required for flagellin-mediated activation of NF-κB.

### Flagellin-mediated activation of NF-κB in intestinal epithelial cells leads to increased and decreased expression of a subset of TLRs

Stimulation of intestinal epithelial cells with *S. typhimurium *or with purified flagellin led to activation of the proinflammatory gene program (Fig. 6C). We wished to examine whether or not expression of TLR genes would also be altered in flagellin stimulated cells. HT29 cells were treated or not with purified flagellin (0.5 μg/ml) or with TNFα (10 μg/ml) and total RNA was isolated from non-treated and treated cells three hours after stimulation and used to make first-strand cDNA. Real-time RT-PCR using gene-specific primers for each of the TLRs (Superarray, Frederick, MD) and first-strand cDNA prepared from non-stimulated or flagellin stimulated cells was used to generate SYBR-green (Perkin-Elmer) labeled DNA products that were detected in an iCycler™ (Bio-Rad). Interestingly, flagellin only mildly activated the expression of TLR2, while expression levels of TLRs 5, 6, 9 and 10 were decreased by 2-fold (Table 2). Contrastingly, TNF stimulation led to increased expression of TLRs 3 and 4 (1.6- and 3.5-fold respectively), while TLRs 2, 9 and 10 were decreased by approximately 2-, 5- and 3-fold respectively. GAPDH expression served as comparative standard.

**Table 2:** Change in TLR mRNA levels following TNFα or FliC stimulation.

	**Fold change**	**Fold change**
**Gene**	**TNFα stimulated**	**FliC stimulated**
TLR1	ND	ND
TLR2	0.6	1.3
TLR3	1.6	0.6
TLR4	3.5	1.1
TLR5	0.9	0.5
TLR6	0.9	0.6
TLR7	M	M
TLR8	ND	ND
TLR9	0.2	0.5
TLR10	0.3	0.5
GAPDH	1.0	1.0

ND None detected by RT^2^PCR

M None detected above level of minus RT control.

Reverse Transcription and Real Time PCR (RT^2^PCR)-RNA was prepared from cells left untreated, stimulated with TNFα (10 ng/ml) or flagellin (1 μg/ml) for 3 h. RT^2^PCR was performed with an iCycler (Bio-Rad) to quantify SYBR-green labeled products generated from PCR products of 1^st ^strand cDNA prepared from TLR1 through TLR10 mRNA, 18S rRNA, and GAPDH mRNA. RT^2^PCR (25 ul reaction volume) was performed with the appropriate primers (SuperArray) in triplicate with HotStart Taq DNA polymerase (SuperArray) at 95°C for 5 min to activate Taq and amplified for 40 cycles (95°C, 30 sec, 55°C, 30 sec, 72°C, 30 sec). RT^2^PCR was performed on the minus RT controls with TLR5 primers to detect DNA contamination. Fold change in mRNA expression was expressed as 2^ΔΔCt^. ΔCt is the difference in threshold cycles for the TLR mRNAs and 18S rRNA. ΔΔCt is the difference between ΔCt non-simulated control and ΔCt stimulated sample.

### TLR5 is expressed in cells that don't respond well to flagellin

This study and others [22,33] have identified TLR5 as the likely TLR through which flagellin activates NF-κB. Previous reports made no determination on the presence or abundance of TLR5 in the cells that they used to ascertain its function [22,33]. We wished to determine if TLR5 protein abundance was absent or greatly decreased in cells that failed to respond or responded poorly to challenge by flagellin. TLR5 abundance in a number of cell lines was examined by immunoblot analysis using a TLR5-specific antibody and compared with the ability of purified flagellin to induce NF-κB DNA binding activity of WCEs prepared from them. Intestinal epithelial cell lines T84 and HT29 were used as was the lung adenocarcinoma cell line A549, the human cervical adenocarcinoma cell line HeLa, the human embryonic kidney cell line expressing large T antigen HEK293T, and the glioblastoma cell line T98G. TLR5 protein was detected in all cell lines examined by immunoblot with TLR5-specific antibody (Fig. 9A). T84 cells exhibited the highest abundance while expression levels of the other cell lines were similar and appeared not to differ by more than two-fold (Fig. 9A). NF-κB DNA binding activity in non-stimulated, TNFα and flagellin stimulated cells was analyzed by EMSA assays of WCEs prepared from each cell type (Fig 9B). HT29 and A549 cells responded strongly to flagellin and to TNFα stimulation while HeLa, 293T and T98G cells responded poorly (HeLa, 293T) or not at all (T98G) to flagellin stimulation. The authenticity of the NF-κB DNA binding complex was determined using p65-specific antibody to supershift the NF-κB DNA:protein complex. It is of interest that some cells that express TLR5 either do not respond at all or do so very poorly. This may be due to either lack of receptor presence at the plasma membrane and intracellular localization, inactivating or detrimental mutations in the TLR5 gene in these cell lines or lack of or low abundance of a required co-receptor or adapter protein (as is the case in some cells for TLR4 and its co-receptor/adapter MD2 [30,53,54]). IL-1 can activate NF-κB DNA binding activity in all of the examined cell lines so it appears that the signaling apparatus downstream of MyD88 to NF-κB is intact.

Recently Muc1 a secreted and membrane bound mucin protein was shown to serve as a receptor that bound *Pseudomonas aeruginosa *and its flagellin, leading to activation of the MAPK pathway [55,56] although NF-κB activity was not examined. We examined the muc1 abundance levels in HT29 (strong flagellin responder), A549 (strong flagellin responder), HeLa, 293T (both weak flagellin responders) and T98G (no flagellin response) to determine if its expression correlated with the activation profile of NF-κB and MAPK in these cells in response to flagellin [55]. Should this be the case then muc1 might serve as a viable co-receptor for TLR5 in propagating activation signals leading to NF-κB activation. We observed that only HT29 cellular proteins gave a strong signal by immunoblot analysis using an muc1-specific antibody while muc1 was barely detectable in the other cell lines (Fig. 9C). These results suggest that muc1 does not serve the role of a TLR5 co-receptor that leads to NF-κB activation and likely plays little to no role activating MAPK pathways in A549 cells where we have observed similar temporal MAPK activation in response to flagellin exposure as we do in HT29 cells (TT and JD, unpublished results). Further examination of muc1's role in HT29 cells in regards to NF-κB and MAPK signaling using siRNA is warranted.

## Discussion

Intestinal epithelial cells at mucosal surfaces serve as innate immune sentinels controlling the innate host defense instruction to the immune effector cells inside the body in response to the external environment [1,2]. Previous studies examining pathogenic *Salmonella *invasion of intestinal epithelial cells demonstrated activation of the proinflammatory gene program and invasion of only a minor portion of the cells [10]. We previously demonstrated that NF-κB is as potently induced in pathogenic *Salmonella .sp *infected cells similar to those treated with the proinflammatory cytokines that are potent NF-κB activators such as TNFα and IL-1β and that this activity was IKK-mediated [3]. Here we examined how bacterial invasion of only a third of the cells could give rise to NF-κB activity profiles consistent with activation of NF-κB in every cell such as the profile TNFα stimulation provides. We found that bacterial infection activates nuclear translocation of p65 (RelA) in nearly all of the intestinal epithelial cells consistent with the hypothesis that a cell surface receptor was recognizing either a soluble product that bacteria were producing, or a bacterial product on the bacteria, or both. We examined bacterial culture broths and found a bacterial product that was protein in composition and when used to challenge intestinal epithelial cells it potently activated NF-κB DNA binding activity (Fig. 2A). We further purified this protein by gel permeation and anion exchange chromatography and found the protein to be flagellin by electrospray ion trap mass spectroscopy (Fig. 2B &2C and Fig. 3). While our studies were in progress, flagellin was identified as being a potent proinflammatory mediator leading to IL-8 production and secretion [16-18]. We demonstrate in this study that flagellin appears to be exclusively responsible for activating NF-κB in intestinal epithelial cells since flagellin mutant strains do not activate NF-κB (Fig. 4) nor lead to their internalization (Fig. 5B). Furthermore, flagellin challenge of intestinal epithelial cells leads to p65 (RelA) nuclear localization in nearly all of the treated cells (Fig. 5B). Transcription factors like activator protein 1 (AP-1) and NF-κB, which are key regulators/activators of the proinflammatory gene program [57,58] are activated by engagement of the MAPK, SAPK and IKK signaling pathways. We demonstrate that the MAPK, SAPK and IKK signaling pathways activation fails to occur in host cells by infection/exposure to *Salmonella *strains devoid of flagellin or products in the culture broths derived from those mutant *Salmonella *strains (Figs. 5A and 6B). We also demonstrate here that combined mutants of both fliC and fljB exhibit a severe lack of invasion (10^-4 ^less than wild-type) and failure to activate stress response signaling, which has not been revealed previously. It is likely that the lack of flagellin production interferes with the functioning of the type III secretion system (TTSS) although flagellin is not known to effect expression of TTSS-required gene products. This hypothesis seems credible since supply of flagellin or bacterial culture components from wild-type *Salmonella *cultures in *trans *to the double fliC^-^/fljB^- ^mutant bacteria fails to compliment their lack of infectivity in gentamycin invsion assays (TT & JD, unpublished observations and see Fig. 5B). In fact, we found the abundance of a subset of Sip and Sop proteins (SipA and SopD) released into the bacterial culture media to be drastically reduced in the flagellin mutant strains used here (TT & JD, unpublished observations). These two proteins have not previously been identified as activators of NF-κB nor are they considered as such here.

The TTSS translocates the *Salmonella *invasion proteins (Sips) and the SopE proteins into the host cell initiating cytoskeletal rearrangements that ultimately lead to bacterial internalization, [11,12,41]. In any event, it is clear that purified flagellin activates a similar cadre of proinflammatory genes as does infection of intestinal epithelial cells with wild-type flagellated *Salmonella*. The temporal expression pattern of these genes was found to be remarkably similar (Fig. 6C) indicating that flagellin-mediated temporal activation of the MAPK, SAPK and IKK signaling pathways can suffice for signaling pathways activated by Sips or SopE and SopE2 and largely recapitulates the temporal activation of key proinflammatory genes as does infection of intestinal epithelial cells with wild-type flagellated *Salmonella*.

The rapid, and potent activation of the MAPK, SAPK and IKK signaling pathways by flagellin was consistent with and indicative of the activation of a cell surface receptor. In this study and in other studies TLR5 has been demonstrated to play an integral role in the recognition of flagellin leading to activation of NF-κB and expression of the IL-8 gene (Fig. 6C) [22,33]. Identification of TLR5 utilized transfection of TLRs 1 – 9 into cell lines which responded poorly to flagellin (this study) or not at all [22,33] and challenging the transfectants with flagellin to identify which TLR responded to this PAMP. Previous studies that identified TLR5 as the receptor for flagellin did not examine the abundance of TLR5 in these cells or account for the lack of TLR5-mediated signaling in response to flagellin [22,33]. We demonstrate here that cells which respond poorly (HeLa and HEK293T) or not at all (T98G) contain TLR5 in at least equivalent abundance as HT29 cells which are highly responsive to flagellin. We propose at least three possibilities to account for this discrepancy, first, this may be due to either lack of TLR5 receptor presence at the plasma membrane and intracellular localization; second, inactivating or detrimental mutations in the TLR5 gene in these cell lines; and lastly, lack of or low abundance of a required co-receptor or adapter protein required for either efficient ligand recognition and/or signaling. These possibilities are currently being investigated. We favor the last possibility since surface biotinylation experiments indicate that TLR5 is present on the cell surface in both flagellin responding cells and in non-responders mentioned above (data not shown). Invocation of the second hypothesis would require inactivating mutations be present in three different cell lines, a highly improbable outcome.

How do the findings presented here correlate with events during a "normal" *Salmonella *infection? We have indicated in this study that defective type III secretion system functioning leads to loss of host cell infectivity and underscores the importance of this system in the normal course of infection. In the *in vivo *setting, polarized epithelial cells express TLR5 on the basolateral surface [48] and flagellin can only reach the receptor either after either breaching the tight junction barrier by physical damage or by loosening of the junctions in response to Sips and Sops delivered into the intestinal epithelial cells by the TTSS or by delivery of flagellin across the intestinal epithelial cell by the bacteria itself [17,59-61]. This scenario would imply the main function of the type III secretion system would be to trigger stress response signaling facilitating invasion and lead to loosening the tight junctions and result in flagellin/ flagellated bacteria to passing through the junctions and infected cells allowing access the basolateral surface and then systemic dispersion. TLR5 on the basolateral surface of the intestinal epithelial cells, in response to flagellin, could then lead to activation of NF-κB and the proinflammatory gene program and host protection. This model is consistent with activation of the proinflammatory gene program observed in response to flagellated *Salmonella sp*. infection in many reports too numerous to cite here and would allow the innate host defense system a fail-safe way to recognize pathogen exposure. In instances where infection of intestinal epithelial cells by naturally occurring non-flagellated *Salmonella *occurs, a strong proinflammatory response would not initially be presented but the *Salmonella *would instead lead to systemic infection as is the case in chickens with *S. galinarum *and *S. pollorum *and result in typhoid-like disease [62]. Infection of chicken epithelial cells does not lead to proinflammatory gene expression by these non-flagellated pathogens but does when infected with *S. typhimurium *or *S. dublin *[62].

Argument for the existence of an additional TLR5 co-receptor/adapter being in limited abundance or absent might be in evidence from the transfection results presented in Table 1 which demonstrated that overexpression of cell surface localized FLAG-tagged TLR5 only resulted in slightly over a two-fold increase in NF-κB reporter gene expression in response to flagellin. If only TLR5 was required for activation of the signaling pathway should not a much more robust response been observed? We have also used DN-TLR5 transfections and NF-κB-dependent reporter gene assays or overexpresssion of DN-TLR5 using recombinant adenoviruses and analysis of resulting NF-κB DNA binding activity in response to flagellin to examine its effectiveness to completely inhibit TLR5-mediated flagellin activation of NF-κB. We have found it difficult to gain more than a fifty-percent reduction in either reporter gene activation or NF-κB DNA binding activity in HT29 cells (TT, AD & JD, unpublished observations). These results suggest that the resting TLR5 signaling complex may be quite stable as has recently been suggested [63]. Should the endogenous TLR5 signaling complex be extremely stable it would therefore be expected that titration of a required pre-stimulus bound adapter or co-receptor away would be inefficient and this is what we have observed. Expression of a DN-MAL (TIRAP), a MyD88-related TLR adapter [64,65] had little to no effect on flagellin-mediated NF-κB activity in transient transfection NF-κB reporter gene assays (TT & JD, unpublished observations). Recently, TLR5 has been shown to bind flagellin [66-68] and that this is likely a direct interaction due to failure of the human TLR5 to respond to a purified flagellin derived from a mouse-specific *Salmonella *strain [68]. These observations still do not preclude the existence of a co-receptor or adapter that is critical for signal transmission. Detailed biochemical characterization of the TLR5 signaling complex will resolve this issue. Muc1, a recently described flagellin interacting membrane protein by virtue of its ability to trigger activation of the MAPK pathway in response to flagellin exposure [55] was considered a viable candidate for such a co-receptor but our observations suggest that it can not serve as the putative TLR5 co-receptor as it is expressed at similar levels in flagellin non-responding cell lines examined here as it is in A549 cells which respond strongly to flagellin and both cell line types express similar levels of TLR5 (Fig. 9).

## Conclusion

In conclusion, our data clearly demonstrates that flagellin can act as the major determinant in activating key stress response signaling pathways and proinflammatory gene program expression in a temporal and qualitative fashion as observed during infection of intestinal epithelial cells by wild-type *Salmonella sp*. that express flagellin, a point that was not well established until this study. In addition, expression of the *fli C *gene appears to play an important role in the proper functioning of the TTSS since mutants that fail to express *fli C *are defective in expressing a subset of Sip proteins and fail to invade host cells. Flagellin added in *trans *cannot restore the ability of the *fli C *mutant bacteria to invade intestinal epithelial cells. Flagellin is "sensed" by TLR5 and in response propagates signaling pathways culminating in potent proinflammatory gene expression. Interestingly we found that TLR5 is expressed in weakly responding and also in some flagellin non-responding cells, 293T, HeLa and T98G cells respectively at levels similar to cells such as HT29 and A549 cells that respond strongly to flagellin and can be found on the cell surface, raising a strong possibility that productive TLR5 signaling may require an additional factor/adaptor other than those already known to be key in the IL-1 signaling pathway, which shares extensive similarities to the TLRs signaling pathways.

## Methods

### Materials

Human tumor necrosis factor alpha (TNFα) and human IL-1 were purchased from R&D Systems (Minneapolis, MN). Tris [hydroxymethyl]aminomethane (Tris) was purchased from Fisher Scientific (Fairlawn, NJ). Fetal calf serum was purchased from US Biotechnologies Inc. (Parkerford, PA). Para-nitro-phenylphosphate (PNPP) was purchased from Aldrich Chemical (Milwaukee, WI). The Polyacrylamide gel electrophoresis (PAGE) supplies: acrylamide, bis-acrylamide, sodium dodecyl sulfate (SDS), TEMED, and ammonium persulfate were purchased from Bio-Rad Laboratories (Hercules, CA). Dulbecco's modified essential medium (DMEM), DMEM:F12, phosphate buffered saline (PBS), glutamine, penicillin G, streptomycin, amphotericin B, and Grace's Insect medium were purchased from Invitrogen (Carlsbad, CA). Luria Broth (LB) was purchased from Becton Dickson and Co (Sparks, MD). The protease inhibitors: aprotinin, bestatin, leupeptin, pepstatin A, and phenylmethylsulfonyl fluoride (PMSF) were purchased from Cal Biochem (La Jolla, CA). Protease inhibitor cocktail contained 10 μg/ml aprotinin, 2.5 μg/ml leupeptin, 8.3 μg/ml bestatin, and 1.7 μg/ml pepstatin A. Phorbol 12-myristate 13 acetate (PMA), N-[2-hydroxyethyl]piperazine-N'-[2-ethanesulfonic acid] (Hepes), anisomycin, and 2-[N-morpholino]ethanesulfonic acid (MES) were purchased from Sigma Chemical (St. Louis, MO). All other reagents were purchased from Sigma Chemical or Fisher Scientific unless stated otherwise.

### Cell culture

HT29 human intestinal (colorectal adenocarcinoma) epithelial cells (ATCC HTB-38), HeLa cervical epithelial adenocarcoma cells (ATCC CCL-2), 293T kidney cells (CRL-11268), A549 lung carcinoma cells (ATCC-185), and T98G glioblastoma cells (ATCC CRL-1690) were cultured in DMEM with 2 mM glutamine, 10% Fetal Calf Serum, 100 Units/ml Penicillin G, and 100 μg/ml Streptomycin at 37°C in a humidified 5% CO_2 _atmosphere. T84 colorectal carcinoma cells (ATCC CCL-248) were cultured in DMEM:F12 with 2 mM glutamine, 5% Fetal Calf Serum, 100 Units/ml Penicillin G, and 100 μg/ml Streptomycin at 37°C in a humidified 5% CO_2 _atmosphere. H5 insect cells (Invitrogen) were cultured in Grace's medium with 2 mM glutamine, 10% Fetal Calf Serum, 100 Units/ml Penicillin G, 100 μg/ml Streptomycin, and 0.25 μg/ml amphotericin B at 28°C. MyD88^-/- ^& TLR2^-/-^/TLR4^-/- ^double knockout cells were obtained from Shizuo Akira and Osamu Takeuchi (Univ. of Osaka, Japan) and grown in DMEM with 2 mM glutamine, 10% Fetal Calf Serum, 100 Units/ml Penicillin G, and 100 μg/ml Streptomycin at 37°C in a humidified 5% CO_2 _atmosphere.

### Bacterial strains

*Salmonella typhimurium *strain SJW1103 (FliC, phase 1 flagellin, stabilized) [69] is a wild-type *Salmonella typhimurium *and can only express the Phase I fliC flagellin, SJW86 (SJW1103 FliC::TN10), and SJW134 (SJW1103 FliC and FljB deletions) were obtained from Robert Macnab (Yale Univ., Conn) and have been described [70]. *Salmonella *serovar dublin strain 2229, strain SE1 (2229 SopE mutant), strain SB2 (2229 SopB mutant), and SE1SB2 (2229 SopE and SopB mutant) were obtained from Edward Galyov (Compton Laboratory, Berkshire, UK) and have been described [14,15]. *Salmonella *strains for stimulation were grown in LB at 37°C without agitation for 16 hours, centrifuged at 6,000 × g for 1 minute, gently washed with PBS, and gently suspended in DMEM to maintain cells with attached flagella.

Plasmid pFM10.1 (ampicillin resistance), encodes a green fluorescent protein (GFP) expressed after the *Salmonella *host is internalized by mammalian cells, obtained from Stanley Falkow (Stanford Univ., Stanford, CA) [10,34] and was transformed into strains SJW1103 and SJW134 by electroporation. Strains containing pFM10.1 were designated SJW1103G and SJW134G.

### Preparation and analysis of Salmonella cell free culture supernatant

Native flagellin was harvested from *S. dublin *2229 or *S. typhimurium *SJW1103. Starter cultures were grown in Luria broth (LB) for 18 hours at 37°C with aeration, diluted 1:5000 in fresh LB, and grown for 12 hours under the same conditions. All subsequent procedures were performed at 4°C. Cells were removed from the medium by centrifugation at 10,000 × g for 5 min and discarded. The supernatant containing flagellin was filtered through a 0.8 micron filter (Millipore, Bedford, MA) to remove residual cells. Supernatant was concentrated 100 fold using an Amicon 100 kiloDalton (kDa) cutoff membrane (Millipore). Initial studies used concentrated culture supernatant from *S. dublin *strain 2229 that was treated with DNase, RNase, Protease K, boiled for 20 min or 100 mM DTT at 37°C for 2 hours and used for stimulation of cultured cells.

Concentrated *S. typhimurium *1103 bacterial culture supernatant was washed 4 times by 1:10 dilution with 50 mM MES, pH 6.0, 50 mM NaCl and re-concentrated. Material not retained by the 100 kDa membrane was discarded. Washed culture supernatant was fractionated by gel permeation or anion exchange chromatography for analysis. For long-term storage, washed culture supernatant was supplemented with protease cocktail and stored at -20°C.

Fractionation by gel permeation chromatography was performed with a Superose 12HR column (Pharmacia) on a Bio-Logic system (Bio-Rad). One-half mililiter of 100× washed supernatant (equivalent of 50 ml original culture supernatant) was separated on the column at 0.4 ml/minute in 50 mM Hepes, pH 7.4, 200 mM NaCl. Fractions (0.5 ml) were collected, and 50 μl was fractionated by SDS-PAGE and stained with Bio-Safe Coomassie (Bio-Rad). Thirty microliters of each fraction was used for stimulation of HT29 cells (60 mm dishes) for 45 min and NF-κB DNA binding activity in the resulting whole cell extracts extracts were assayed by EMSA. The column was standardized with catalase (232 kDa), aldolase (158 kDa), abumin (67 kDa), ovalbumin (43 kDa), and Chymotripsinogen A (25 kDa), all obtained from Amersham-Pharmacia.

Fractionation by anion exchange chromatography was performed with Poros HQ matrix (2 ml column, PerSeptive Biosystems, Farmingham, MA) on a Bio-Logic system. Five mililiters of 100× washed supernatant (equivalent of 500 ml original culture supernatant) was separated at 1 ml/minute in 50 mM Hepes, pH 7.4, and a NaCl gradient from 50–500 mM. Fractions were collected and 5 μl of each fraction was examined by 10% SDS-PAGE. Proteins were fractionated on duplicate 10% SDS-PAGE precast gels (BioRad). One gel was stained with Bio-Safe Coomassie (Bio-Rad) and the protein bands were isolated for Mass Spectroscopy analysis (CCF Mass spectroscopy core facility) from the other identical non-stained gel, by electro-elution with a whole gel eluter (Bio-Rad) and SDS was removed with SDS-Out (Pierce, Rockland, IL) per the manufacturers directions. Proteins isolated from bands B1 to B6 were acetone precipitated by addition of 20 μg Aprotinin and 5 μg of BSA to each eluted fraction, ice-cold acetone (-20°C) was added to 80%, mixed well and precipitated overnight at -20°C. Proteins were pelleted by centrifugation at 14,000 × g in the cold for 30 min, acetone/liquid was removed and the pellets washed 2× with 1 ml acetone (-20°C). After removal of the acetone, protein pellets were air dried and then resuspended and denatured in 5 μl of 6 M guanidinium hydrochloride (Gu-HCl) at room temperature for 30 min. Resuspended proteins were two-fold serially diluted in DMEM to a final Gu-HCl concentration of 55 mM to renature the proteins. Two hundred fifty microliters of individual renatured proteins/DMEM were added per ml to HT29 cells (60 mm dishes) and whole cell extracts were prepared 45 min after stimulation and were assayed for NF-κB DNA binding activity by EMSA.

### Purification of flagellin (purified flagellin)

The washed and concentrated culture supernatant from *S. typhimurium *1103 containing flagellin was boiled for 20 minutes and precipitants removed by centrifugation at 15,000 × g. The supernatant containing flagellin was diluted 1:2 with 50 mM MES, pH 6.0, 50 mM NaCl and mixed with 2 ml Poros SP cation exchange matrix (PerSeptive Biosystems) per 1 liter of original culture. The Poros SP matrix was prepared as a 50% slurry and equilibrated with 50 mM MES, pH 6.0. The flagellin preparation and matrix were mixed on a roller at 12 to 14 RPM for 2 hours. The matrix along with bound contaminants was removed by filtration through a 0.85 micron filter and discarded, flagellin failed to bind to the cation exchange matrix at pH 6.0 and eluted in the flowthrough and was collected.

The pH of the flowthrough was adjusted by five-fold dilution of the sample with 50 mM Hepes, pH 7.8, 50 mM NaCl, and loaded onto a Poros HQ anion exchange column (2 ml column, PerSeptive Biosystems) equilibrated with 50 mM Hepes, pH 7.4, 50 mM NaCl. The column was washed with 2 volumes 50 mM Hepes, pH, 7.4, 50 mM NaCl, and eluted with a 10 column volume linear gradient of 50–500 mM NaCl in 50 mM Hepes, pH 7.4. Flagellin eluted from the column between 200–275 mM NaCl. Fractions containing flagellin were pooled and concentrated. The preparation was determined to be pure by electrophoresis of 5 μg protein by SDS-PAGE and stained with Bio-Safe Coomassie (Bio-Rad). Samples were stored at -80°C in 50 mM Hepes, pH 7.4, approx 225 mM NaCl, 10% glycerol and protease cocktail. A 4 liter preparation of culture supernatant yielded 2 mg purified flagellin.

### In-gel tryptic digestion and protein identification by LC-MS

Gels were fixed and stained (Bio-Safe Blue, BioRad). All of the following procedures were performed by the CCF Mass spectroscopy core facility. Excised gel bands were reduced (100 mM DTT), and alkylated (100 mM iodoacetamide). Proteins in the gel bands were digested with modified trypsin (Promega, 20 μg/mL) with an overnight incubation at 37°C. Tryptic peptides were extracted from the gel with 50% acetonitrile, 0.1% acetic acid, concentrated in a SpeedVac (Thermo Savant) to remove acetonitrile, and reconstituted to 20 uL with 0.1% acetic acid. Extracted peptides were subjected to reversed phase (50 uM ID packed with Phenomenex Jupiter C18, 6 cm capillary column) liquid chromatography (2%–70% solvent B; Solvent A, 50 mM acetic acid, aqueous, Solvent B acetonitrile), coupled to a Finnigan LCQ DECA ion trap mass spectrometer for peptide sequencing, as described [38].

### Preparation of GST-IκBa1-54 and GST-cJUN1-79 kinase substrates

IκBα amino acids 1 to 54 fused to GST or cJUN amino acids 1–79 fused to GST were prepared as previously described [37-39] and stored in kinase buffer (20 mM Hepes, pH 7.6, 10 MM MgCl2, 10 mM NaCl, 2 mM beta-glycerophosphate, 10 mM PNPP).

### Preparation of cells for microscopy

HT29 cells for microscopic examination were grown in 6 well plates on sterile cover slips to a density of 50–75%. Cells were stimulated as described above. After stimulation, cover slips with HT29 cells were washed 2 times with ice cold PBS and fixed with 4% w/v formalin at room temperature for 20 minutes. Cells were washed 4 times with PBS prior to mounting for visualization of *Salmonella *invasion. Cover slips were mounted with Vectashield mounting medium with DAPI (Vector Laboratories, Burlingame, CA), and cover slips sealed to slides.

Cells for antibody staining were treated with absolute methanol for 20 minutes following formalin fixation, then washed 3 times with PBS supplemented with 0.1% BSA (PBSB) and used directly or stored in the cold after azide was added to 0.02%. For p65(RelA) localization, cells on coverslips were blocked for 1 h at 37°C with PBS supplemented with 1% BSA. The PBSB was removed, washed once with PBSB and coverslips were placed cell-side down onto 150 μl of p65 antibody (Zymed, South San Francisco, CA) diluted 1:1500 in PBSB on a square of parafilm and placed in a humidified chamber at 37°C for 1.5 h. Coverslips were removed and placed cell-side up in 6-well dishes and washed 3 × 5 min with PBSB. Coverslips were then removed and placed cell-side down onto 150 μl of FITC-labeled donkey anti-rabbit secondary antibody (Jackson Immunoresearch Laboratories, West Grove, PA) (1:300 in PBSB) on a square of parafilm and placed in a humidified chamber at 37°C for 1.5 h. Coverslips were removed and placed cell-side up in 6-well dishes and washed 5 × 5 min with PBSB, removed and placed cell-side down onto slides mounted with Vectashield (Vector Laboratories, Burlingame, CA) with DAPI and then sealed. NF-κB localization was determined by indirect immunofluorescence. Samples were observed on a Leica DMR upright microscope (Leica Microsystems Inc., Heidelberg, Germany) at 400× with oil immersion and equipped with FITC and UV filters. Images were collected with a MicroMax RS camera (Princeton Instruments Inc., Princeton, NJ), and Image Pro plus, version 4.5, software (Media Cybermetics Inc., Carlsbad, CA). Color enhancements were performed with Image Pro plus software. Visible light plus color overlays for Fig. 1 and Fig. 5B were performed with MetaMorph Software (Universal Imaging Corp., Downington, PA).

### Bacterial infection and cell stimulation

Mouse embryo fibroblasts (MEFs) or HT29 cells were grown in DMEM as above to a density of 90% prior to stimulation. All cells were washed with warm PBS and supplemented with DMEM without serum or antibiotics in preparation for stimulation. Cells were stimulated with; 10 ng/ml TNFα, 1 μg/ml flagellin unless specified otherwise, 20 μg/ml Anisomycin, 12.5 ng/ml PMA, or 10^8 ^*Salmonella*/ml at 37°C for desired times and extracts prepared as below. Cells harvested beyond one hour were washed with warm PBS and supplemented with warm DMEM, 2 mM glutamine, and 200 ug/ml gentamycin after 1 hour and returned to 37°C until extract preparation desired.

### Whole cell extract preparation

Cells were washed with ice-cold PBS and all subsequent steps carried out at 4°C or on ice. Cells were scraped from the dish in ice-cold PBS, and collected by centrifugation at 1000 × g for 1 minute. Cells were lysed by suspension in 50 mM Tris-HCl, pH 7.6, 400 mM NaCl, 25 mM beta-glycerol phosphate, 25 mM NaF, 10 mM PNPP, 10 % glycerol, 0.5 mM sodium orthovanadate, 0.5% nonidet-40 (NP-40), 5 mM benzamidine, 2.5 mM metabisulfite, 1 mM PMSF, 1 mM DTT and protease inhibitor cocktail as described [3].

### Electromobility shift assays (EMSA)

NF-κB DNA binding assays were carried out as previously described [3,35,38]. Anti-p65 antibody (Zymed, South San Francisco), anti-p50 antibody (Santa Cruz Biotechnologies, Santa Cruz, CA), and anti-STAT3 antibody (Santa Cruz) were used for EMSA supershifts.

### Invasion assay

HT29 cells, 90–95% confluent in 35 mm round dishes, were prepared for stimulation as above and treated with a 1 ml suspension of Salmonella SJW1103 or SJW134 or left untreated in triplicate as above. After one hour, HT29 cells were washed 4× with warm PBS, supplemented with warm DMEM, 2 mM glutamine, and 200 μg/ml gentamycin, and incubated at 37°C for 4 hours. Cells were then harvested as above and lysed by suspension in 1 ml sterile distilled water. Ten-fold serial dilutions were prepared in PBS and 100 μl of each dilution was plated on LB agar plates and grown at 37°C for 20 hours. Colonies were counted and averaged.

### Kinase assays

Whole cell extracts (250 μg) were supplemented with 150 μl of Buffer A (20 mM Hepes, pH 7.9, 20 mM beta-glycerophosphate, 10 mM NaF, 0.1 mM orthovanadate, 5 mM PNPP, 10 mM 2-mercaptoethanol, 0.5 mM PMSF, and protease inhibitor cocktail), and immuno precipitation kinase assays carried out as described [3] using either IKKα monoclonal antibody (PharMingen – Becton Dickson), anti-JNK1 (Santa Cruz Biotechnologies, Santa Cruz, CA), or anti-hemagglutinin (HA) epitope antibody (Covence Antibodies, Princeton, NJ) as indicated. Protein G immunopellets were collected by centrifugation at 500 × g for 30 sec, washed 3 times with Buffer B (Buffer A plus 250 mM NaCl), and one time with Buffer C (Buffer A plus 50 mM NaCl and 10 mM MgCl_2_). Immunopellets were resuspended in 30 μl Kinase buffer with 0.1 mM orthovanadate, 50 μM "cold" ATP, 5 μCi γ-^32^P-ATP, 2 mM DTT, and 2 μg of soluble GST-IκBα1–54 or GST-cJUN1-79, and incubated at 30°C for 30 minutes. Reactions were stopped by the addition of 15 μl 4× SDS-PAGE loading buffer, heated at 95°C for 5 minutes, and resolved on 10% SDS-PAGE gels by standard procedures. Gels were rinsed, stained with Bio-Safe Coomassie (Bio-Rad) to visualize protein bands, rinsed, photographed then dried and exposed to Kodak X-OMAT AR film (Eastman Kodak Co., Rochester, NY) to detect substrate phosphorylation.

### Immunoblotting

Protein samples (40 μg) were resolved by SDS-PAGE on a 10% acrylamide gels by standard procedures, and proteins transferred to PVDF membrane (Millipore) and probed with antibodies as described [3]. Membranes were washed 3× briefly with TBST, incubated with a 1:1000 dilution (1:800 for anti-TLR5) of the primary antibody in TBST, 1% non-fat milk for 1 hour, washed 3 × 5 min with TBST, and then incubated with a 1:2000 dilution of the appropriate HRP-conjugated secondary antibody in TBST, 0.5% non-fat milk for 1 hour. Primary antibodies used were: anti-IKKα/β (H-470, Santa Cruz), anti-JNK1, anti-ERK2 (K-23, Santa Cruz), anti-phospho-ERK (E-4, Santa Cruz), anti-p38MAPK (Cell Signaling Technologies, Beverly, MA), anti-phosopho-p38MAPK (Cell Signaling), anti-TLR5 (H-127, Santa Cruz), anti-muc1 (H-295, Santa Cruz) and anti-actin (C-11, Santa Cruz). Secondary antibodies used were: anti-mouse IgG HRP conjugate (Amersham-Pharmacia), anti-rabbit IgG HRP conjugate (Amersham-Pharmacia), anti-goat IgG-HRP conjugate (Santa Cruz). HRP activity was detected by ECL (Amersham-Pharmacia) as per manufacturers instructions, on Kodak X-OMAT AR film.

### Construction of dominant-negative TLRs

All DN-TLRs were constructed using PCR. The universal 5' primer consisted of a 5'KPN I restriction site followed by sequences encoding the kozak sequence, translational start site, and preprotrypsin leader sequence of pCMV-1 (Sigma) that all the wild-type TLRs were initially cloned into. The 3' anti-sense (AS) primers were human TLRgene-specific primers (sequences available upon request) that created a stop codon immediately after a conserved tryptophan in Box 9 of the TLR TIR homology domain according to Bazan [71], thus creating carboxy terminus deletions. The 5' end of the AS primer contained a number of convenient restriction sites to allow directional cloning. PCR was performed with turbo-Pfu polymerase (Stratagene, La Jolla, CA) using standard procedures on individual wild-type TLR pCMV-1 plasmid DNAs (5 ng each, kind gifts of R. Medzhitov, Yale Univ. and R. Ulevitch, TSRI) [48,49] with the 150 ng each of the universal 5' sense primer and individual gene-specific TLR 3' primers. PCR products were cleaned-up with PCR cleanup kit (Qiagen, Germany) digested with appropriate restriction enzymes, gel purified and then ligated into the mammalian expression vector pCDNA3.1 (Invitrogen). Positive clones were sequenced to verify the mutations and tested for expression in transient expression assays and detected on immunoblots by probing with anti-FLAG M2 monoclonal antibody (Sigma). All wild-type and DN-TLR alleles are amino terminus FLAG epitope-tagged.

### Transfections

HT29 cells were transfected with Lipofectamine Plus (Invitrogen) as previously described [3]. In transfections monitoring reporter gene expression, transfections were performed at least three times in 6 well dishes in triplicate with the total DNA mass kept constant at 4 μg (2 μg effector plasmid DNA, 100 ng 2× NF-κB Luc reporter gene, 50 ng pRL-TK, a thymidine kinase promoter driven *Renilla *luciferase normalization reporter and 1.85 μg pCDNA3.1 plasmid DNA as bulk filler DNA) and fire-fly luciferase expression was normalized to *Renilla *luciferase expression using the dual-luciferase assay (Promega, Madison, WI). Fold inductions were calculated and values between experiments did not vary more than 15%, a representative experiment is presented. Transfection of 293T cells was performed with lipofectamine 2000 (Invitrogen) in 6-well dishes in triplicate as per the manufacture's protocol. TLR expression plasmids were added at 2 μg/well, and NF-κB and normalization control plasmids were as above with HT29 cells and pCDNA3.1 plasmid DNA as bulk filler DNA to a final DNA mass of 4 μg/well. Fold inductions were calculated and values between experiments (N of 3) did not vary more than 10%, a representative experiment is presented.

### Real Reverse Transcription and Real Time PCR (RT^2^PCR)

Cells (N = 3) were stimulated 3 hours at 37°C with TNFα or FliC or left untreated and harvested for total RNA isolation. Total cellular RNA was extracted from cells with Trizol reagent (Invitrogen) [3] and reverse transcribed with ReactionReady first strand cDNA synthesis kit (SuperArray Bioscience Corp., Fredrick, MD). RNA (2.5 ug per 20 ul reaction) was reverse transcribed using random primers and Moloney murine leukemia virus reverse transcriptase per manufacturer specified conditions. Controls without reverse transcriptase (minus RT) was also generated for each RNA sample. RT^2^PCR was performed with an iCycler (Bio-Rad) to quantify TLR1 through TLR10 mRNA, 18S rRNA, and GAPDH mRNA. RT^2^PCR (25 ul reaction volume) was performed with the appropriate primers (SuperArray) per manufacturers instructions in triplicate with HotStart Taq DNA polymerase (SuperArray) at 95°C for 15 min to activate Taq and amplified for 40 cycles (95°C, 30 sec, 55°C, 30 sec, 72°C, 30 sec). RT^2^PCR was performed on the minus RT controls with TLR5 primers to detect DNA contamination. Real-time PCR analysis was performed using SYBR-green (Perkin-Elmer) according to manufacture's instructions with the specific primer pairs indicated above and primer pairs for 18S ribosomal RNA as reference RNA (Classic 18S primer pairs – Ambion Inc). Cycle time (Ct) was measured using the iCycler™ and its associated software (Bio-Rad). Relative transcript quantities were calculated by the ΔΔCt method using 18S ribosomal RNA as a reference amplified from samples using the Classic 18S primer pairs from Ambion, Inc (Austin, TX). Normalized samples were then expressed relative to the average ΔCt value for untreated controls to obtain relative fold-change in expression levels. Fold change in mRNA expression was expressed as 2^ΔΔCt^. ΔCt is the difference in threshold cycles for the TLR mRNAs and 18S rRNA. ΔΔCt is the difference between ΔCt non-simulated control and ΔCt stimulated sample. Values for fold-induction varied less than 5% among replicates.

## Abbreviations

The abbreviations used are: FBS, fetal bovine serum; IL-1, interlukin-1, SDS, sodium dodecyl sulfate; PAGE, polyacrylamide gel electrophoresis; EMSA, electromobility shift assay; IB, immunoblot; KA, kinase assay; GST, glutathione S-transferase; PBS, phosphate-buffered saline; TNFα, tumor necrosis factor α; NF-κB, nuclear factor kappa B; IKK, Ikappa B kinase; IκB, Ikappa B; PCR, polymerase chain assay; RT-PCR, reverse transcription polymerase chain assay; Gu-HCl, guanidinium hydrochloride ; MAPK, mitogen activated protein kinase; SAPK, stress-activated protein kinase; ERK, extracellular regulated kinase; TLR, toll-like receptor; DN, dominant-negative; JNK, Jun N-terminal kinase; AP-1, activator protein-1; MEF, mouse embryo fibroblast; WCE, whole cell extract; IEC, intestinal epithelial cell; MCP1, macrophage chemoattractant protein 1; TTSS, type III secretion system; Sip, *Salmonella *invasion protein; PMA, phorbol 12-myristate 13 acetate; PNPP, para nitrophenyl phosphate; TK, thymidine kinase; BF, bright field; NP-40, nonidet-40; NRS, normal rabbit serum; IN, input; Ct, cycle time.

## Authors' contributions

TT and AD initiated the study and performed the majority of the experiments and contributed equally and were assisted by NK and JL. MD constructed a number of DN-TLRs and JD developed the study, provided funding support, oversaw the project and also constructed a number of mutant TLRs.
